# Correction: Angiotensin II receptor blocker or angiotensin-converting enzyme inhibitor use and COVID-19-related outcomes among US Veterans

**DOI:** 10.1371/journal.pone.0251897

**Published:** 2021-05-13

**Authors:** The PLOS ONE Staff

The twenty-eighth author’s name is spelled incorrectly. The correct name is: Weiming Xia. The publisher apologizes for the error.
