# Supplementary material for: Angiotensin II receptor blocker or angiotensin-converting enzyme inhibitor use and COVID-19-related outcomes among US Veterans
Source: PLoS One. 2021 Apr 23;16(4):e0248080. doi: 10.1371/journal.pone.0248080 (PMC8064574; doi:10.1371/journal.pone.0248080)
Supplement: S1 File — (DOCX) [file pone.0248080.s001.docx]

**S1 File.**

**Supplemental Material for:**

**Angiotensin II receptor blocker or angiotensin-converting enzyme inhibitor use and COVID-19-related outcomes among US Veterans**

Catherine G. Derington, PharmD, MS;^a^ Jordana B. Cohen, MD, MSCE;^b,c^ April F. Mohanty, MPH, PhD;^d,e^ Tom H. Greene, PhD;^a^ James Cook, MS;^a,d^ Jian Ying, PhD;^a,d^ Guo Wei, MS;^a,d^ Jennifer S. Herrick, MS;^a,d^ Vanessa W. Stevens, PhD;^a,d^ Barbara E. Jones, MD, MS;^d,e^ Libo Wang, MD;^d^ Alexander R. Zheutlin, MD, MS;^e^ Andrew M. South, MD, MS;^f,g^ Thomas C. Hanff, MD, MSCE;^h^ Steven M. Smith, PharmD, MPH;^i^ Rhonda M. Cooper-DeHoff, PharmD, MS;^i,j^ Jordan B. King, PharmD, MS;^a,d,k^ G. Caleb Alexander, MD;^l^ Dan R. Berlowitz, MD, MPH;^m^ Faraz S. Ahmad, MD, MS;^m^ M. Jason Penrod, MD;^e^ Rachel Hess, MD, MS;^a,e^ Molly B. Conroy, MD, MPH;^a,e^ James C. Fang, MD;^e^ Michael A. Rubin, MD, PhD, MS;^e^ Srinivasan Beddhu, MD;^e^ Alfred K. Cheung, MD;^e^ Weiming Xia, PhD;^n,o^ William S. Weintraub, MD;^p^ Adam P. Bress, PharmD, MS^a,d,e^

1. Department of Population Health Sciences, Division of Health System Innovation and Research, University of Utah School of Medicine, Salt Lake City, UT
2. Department of Medicine, Renal-Electrolyte and Hypertension Division, Perelman School of Medicine at the University of Pennsylvania, Philadelphia, PA
3. Department of Biostatistics, Epidemiology, and Informatics, Perelman School of Medicine, University of Pennsylvania, Philadelphia, PA
4. George E. Wahlen Department of Veterans Affairs Medical Center, Salt Lake City, UT
5. Department of Internal Medicine, University of Utah School of Medicine, Salt Lake City, UT
6. Department of Pediatrics, Section of Nephrology, Brenner Children’s Hospital, Wake Forest School of Medicine, Winston Salem, NC
7. Division of Public Health Sciences, Department of Epidemiology and Prevention, Wake Forest School of Medicine, Winston Salem, NC
8. Department of Medicine, Division of Cardiology, Perelman School of Medicine, University of Pennsylvania, Philadelphia, PA
9. Department of Pharmacotherapy and Translational Research, University of Florida College of Pharmacy, Gainesville, FL
10. Department of Medicine, University of Florida, College of Medicine, Gainesville, FL
11. Institute for Health Research, Kaiser Permanente Colorado, Aurora, CO
12. Department of Epidemiology, Johns Hopkins Bloomberg School of Public Health, Baltimore, MD
13. Department of Cardiology, Northwestern University Feinberg School of Medicine, Chicago, IL
14. Department of Pharmacology and Experimental Therapeutics, Boston University School of Medicine, Boston, MA
15. Edith Nourse Rogers Memorial Veterans Hospital, Bedford, MA
16. MedStar Washington Hospital Center, Washington, DC

**Table of Contents**

**Supplemental Methods (page 4-5)**

- Data sources
- SARS-CoV-2 testing
- Multiple imputation
- Subgroup analyses
- Sensitivity analyses

**Supplemental Tables (pages 6-42)**

- Supplemental Table S1: Variables used to define the study population, baseline covariates, exposures, and outcomes.
- Supplemental Table S2: List of antihypertensive medications by class.
- Supplemental Table S3: Covariates included in the estimation of the propensity scores.
- Supplemental Table S4: Baseline characteristics of outpatient Veterans with treated hypertension without compelling indications for an ARB or ACEI who are current users of an ARB/ACEI-based antihypertensive regimen vs. non-ARB/ACEI-based antihypertensive regimen, before and after propensity score weighting.
- Supplemental Table S5: Baseline characteristics of outpatient Veterans with treated hypertension who are current users of ARB vs. ACEI, before and after propensity score weighting.
- Supplemental Table S6: Baseline characteristics of inpatient Veterans with treated hypertension without compelling indications for an ARB or ACEI who are current users of an ARB/ACEI-based antihypertensive regimen vs. non-ARB/ACEI-based antihypertensive regimen, before and after propensity score weighting.
- Supplemental Table S7: Baseline characteristics of inpatient Veterans with treated hypertension who are current users of ARB vs. ACEI, before and after propensity score weighting.
- Supplemental Table S8: Matching weight adjusted incidence rates and hazard ratios for all-cause mortality among outpatient Veterans with treated hypertension who are current users of ARB/ACEI-based antihypertensive regimen vs. non-ARB/ACEI-based antihypertensive regimen in subgroups: Matching weight adjusted incidence rates
- Supplemental Table S9: Matching weight adjusted incidence rates and hazard ratios for all-cause hospitalization and all-cause mortality among outpatient Veterans with treated hypertension who are current users of an ARB vs. ACEI in subgroups: Matching weight adjusted incidence rates
- Supplemental Table S10: Matching weight adjusted incidence rates and hazard ratios for all-cause hospitalization Veterans hospitalized for COVID-19 who are current users of an an ARB vs. ACEI in subgroups: Matching weight adjusted incidence rates
- Supplemental Table S11: Hazard ratios for all-cause hospitalization and all-cause mortality among outpatient Veterans with treated hypertension, by covariate adjustment strategy.
- Supplemental Table S12: Hazard ratios for the primary and secondary outcomes among inpatient Veterans with treated hypertension, by covariate adjustment strategy
- Supplemental Table S13: Matching weight adjusted incidence rates and hazard ratios for the primary and secondary outcomes among outpatient Veterans with treated hypertension who are current users of ARB/ACEI-based antihypertensive regimen vs. non-ARB/ACEI-based antihypertensive regimen, by varying the definition of the primary medication exposure.
- Supplemental Table S14: Matching weight adjusted incidence rates and hazard ratios for the primary and secondary outcomes among outpatient Veterans with treated hypertension who are current users of an ARB vs. ACEI, by varying the definition of the primary medication exposure.
- Supplemental Table S15: Matching weight adjusted incidence rates and hazard ratios for the primary and secondary outcomes among inpatient Veterans with treated hypertension who are current users of an ARB vs. ACEI, by varying the definition of the primary medication exposure.
- Supplemental Table S16: One or more inpatient encounter for negative control outcomes among outpatient Veterans with treated hypertension, matching weight adjusted.
- Supplemental Table S17: One or more inpatient encounter for negative control outcomes among inpatient Veterans with treated hypertension, matching weight adjusted.

**Supplemental Figures (pages 43-54)**

- Supplemental Figure S1: Index date identification period and collection of variables in outpatients (Panel A) and inpatients (Panel B).
- Supplemental Figure S2: Distribution of propensity scores among outpatient Veterans who are SARS-CoV-2 positive between current users of an ARB/ACEI-based antihypertensive regimen vs. non-ARB/ACEI-based antihypertensive regimen (Panel A), and current users of an ARB vs. ACEI (Panel B).
- Supplemental Figure S3: Distribution of propensity scores among inpatient Veterans between current users of an ARB/ACEI-based antihypertensive regimen vs. non-ARB/ACEI-based antihypertensive regimen (Panel A) and current users of an ARB vs. ACEI (Panel B).
- Supplemental Figure S4: Flow diagrams for inclusion of outpatient (Panel A) and inpatient (Panel B) Veterans.
- Supplemental Figure S5: Balance of patient characteristics before and after propensity score weighting.
- Supplemental Figure S6: Matching weight-adjusted cumulative hazard curves for all-cause hospitalization, all-cause mortality, and ICU admission among outpatient Veterans who are current users of an ARB/ACEI-based antihypertensive regimen vs. non-ARB/ACEI-based antihypertensive regimen (Panels A through C) and ARB vs. ACEI (Panels D through F) who are SARS-CoV-2 positive.
- Supplemental Figure S7: Matching weight-adjusted cumulative hazard curves for ICU admission, mechanical ventilation, and dialysis among inpatient Veterans who are current users of an ARB/ACEI-based antihypertensive regimen vs. non-ARB/ACEI-based antihypertensive regimen (Panels A through C) and ARB vs. ACEI (Panels D through F) who are SARS-CoV-2 positive.

**Supplemental References (page 55)**

Supplemental Methods

*Data Sources*

We obtained mortality data from the Vital Status File and pharmacy data from Managerial Cost Accounting, Pharmacy Benefits Management, and the Corporate Data Warehouse.

*SARS-CoV-2 Testing*

During the study period, SARS-CoV-2 tests were performed using nasopharyngeal swabs collected at internal and external facilities. Positive tests reflect Veterans who were positive for SARS-CoV-2 on polymerase chain reaction assays authorized for emergency use at state public health and commercial laboratories.

*Multiple imputation*

To account for missing data we used multiple imputation with chained equations to provide 10 imputed datasets. Within each imputed dataset, we performed bootstrap resampling(2) with 2,000 bootstrap samples to generate means and 95% confidence intervals (CIs). We pooled results across the 10 imputed data sets using Rubin’s formulae (3, 4).

*Subgroup analyses*

All analyses, including fitting logistic regression models to estimate PS, were repeated in subgroups of Veterans according to age, sex, race-ethnicity, body mass index, and the number of antihypertensive medication classes being taken. To test for the difference between subgroups, 200 bootstrapping iterations were performed to obtain the means (M) and covariance (V) of the regression coefficients of subgroups for each of subgroup variables. The p value for the contrast of the means (M) were then calculated based on the null multivariate normal distribution with means 0 and covariance V.

*Sensitivity Analyses*

Covariate adjustment strategies:

Using Cox regression, we presented results using multiple covariate adjustment strategies:

- - 1. Unadjusted (i.e., “crude”): no covariate adjustment.
    2. Multivariable-adjusted: adjustment for all the covariates included in the estimation of the propensity score (PS).
    3. PS-adjusted: Deciles of the PS were added as covariates.
    4. PS stratification: We estimate stratum-specific treatment effects within deciles of the PS. We produced and reported a pooled estimate across strata to estimate an overall treatment effect via the method of Imbens(1).
    5. PS matching: 1:1 PS matched using a greedy algorithm and nearest-neighbor matching with a caliper size equal to 0.2 of the SD of the logit of the PS without replacement.

6. Inverse probability of treatment weighting (IPTW): Weighted using stabilized, traditional IPTW without trimming or truncation.

Alternate definitions of the medication exposure window:

We repeated all analyses among the cohorts, varying the main medication exposure definitions as below, separately. Patients had to have at least one fill in the 104-day pre-index period AND:

1. Two or more pharmacy fills in the 194-day pre-index period;
2. Three consecutive pharmacy fills in the 379-day pre-index period;
3. Pharmacy fill dispensed in the pre-index period with a days’ supply that met or exceeded the index date
4. At least one inpatient medication administration for the same antihypertensive medication on or after the index date (inpatient cohort only).

**Supplemental Tables**

| Supplemental Table S1: Variables used to define the study population, baseline covariates, exposures, and outcomes. | |
| --- | --- |
| **Variable** | **Definition** |
| *Study populations* |  |
| Index date | Date of the first positive SARS-CoV-2 test (outpatient) or admission date of the first hospitalization after the first SARS-CoV-2 positive test (inpatient). |
| SARS-CoV-2 positive test | Veterans with a positive SARS-CoV-2 test between January 19, 2020, to October 15, 2020 will be identified using the VA’s official SARS-CoV-2 phenotype definition, which incorporates testing external to the VA. Patients identified as “VA confirmed” and “VA probable” will be categorized as SARS-CoV-2 positive. The first positive date in this date range will be used. |
| SARS-CoV-2-positive outpatients | Veterans with a positive SARS-CoV-2 test will be identified as above. Then, to ensure that participants are accurately classified as outpatients, patients will be excluded if they have been hospitalized within 7 days before the date of the positive test. |
| Hypertension(5) | Any of the following using all available claims prior to the index date (including the index date):   1. ICD-9 codes:    1. At least 1 inpatient claim with a discharge diagnosis code (any position) of 401.x, 403.0x, 403.1x, 403.9x.    2. ≥2 outpatient claims with a diagnosis code (any position) of 401.x, 403.0x, 403.1x, 403.9x at least 30 days apart. 2. ICD-10 codes:    1. 1 inpatient claim with a discharge diagnosis code (any position) of I10, I12.0, I12.9.   ≥2 outpatient claims with a diagnosis code (any position) of I10, I12.0, I12.9 at least 30 days apart. |
| Treated hypertension | - 1. Having hypertension, as defined above, and currently taking antihypertensive medication, as defined as a pharmacy fill for an antihypertensive medication within 90 days prior to the index date (see Supplemental Table S2 for medication classes). |
| Compelling indication for ARB or ACEI | Individuals with diabetes, chronic kidney disease, heart failure with reduced ejection fraction, coronary heart disease, or history of stroke, as defined below, will be excluded from the cohort samples for the comparison of ARB/ACEI-based antihypertensive regimen vs. non-ARB/ACEI-based antihypertensive regimen. |
|  |  |
| *Baseline covariates* |  |
| Age | Age of Veterans calculated on the index date based on their date of birth. |
| Sex | Male or female |
| Race-ethnicity | Non-Hispanic Black, Non-Hispanic White, Hispanic, Asian, and Other/Missing |
| Area-level income, USD | Using ZIP and FIPS codes based on data obtained from the American Community Survey. The variable ZCTA was matched to ZIP code in the American Community Survey. If ZCTA and ZIP did not match, then FIPS code (county level) from the American Community Survey was used. |
| Veterans Integrated Service Network | Defined by receipt of care in one of the 23 VISNs, categorized into 4 regions after data query: Northeast, Southeast, Continental, and Pacific according to the VA regional offices map.(6) The Northeast region is comprised of VISNs 1, 2, 4, 5, 10, and 12. The Southeast region is comprised of VISNs 6, 7, 8, 9, and 16. The Continental region is comprised of VISNs 15, 17, 18, 19, and 23. Finally, the Pacific region is comprised of VISNs 20, 21, and 22. |
| Current Tobacco Use | Any of the following within one year prior to the index date (including the index date):   1. ICD-9 codes:    1. ≥1 hospitalization with a discharge diagnosis code (any position) of tobacco use of 305.1, 649.0x, 989.84, or V15.82) in any discharge position.    2. ≥1 physician evaluation and management visit with a diagnosis code (any position) of tobacco use of 305.1, 649.0x, 989.84, or V15.82) in any discharge position. 2. ICD-10 codes:    1. ≥1 hospitalization with a discharge diagnosis code (any position) of tobacco use of F17.200, F17.201, F17.210, F17.211, F17.220, F17.221, F17.290, F17.291, or Z87.891) in any discharge position.    2. ≥1 outpatient visit with a diagnosis code (any position) of tobacco of F17.200, F17.201, F17.210, F17.211, F17.220, F17.221, F17.290, F17.291, or Z87.891) in any discharge position. 3. ≥1 hospitalization with a discharge diagnosis code or physician evaluation and management visit of tobacco use with a CPT code (any position) of 99406, 99407, G0436, G0437, G9016, S9453, S4995, G9276, G9458, 1034F, 4004F, 4001F. 4. ≥1 pharmacy claim for nicotine or varenicline in the 379 days before the index date (including the index date). |
| Insurance type | Coded as government (Medicare, Medicaid), private (all insurance external to Medicare/Medicaid), none, and unknown. |
| Priority group status | Coded as 1 through 9 or multiple. |
| Height | Height (in m) on the index date or the date closest to the index date during the one-year pre-index period. Retain values that are within 3 SD of the mean of all heights in the cohort. For Veterans without observations within 3 SD of the mean, other values will be accepted as valid if there are at least 2 identical observations. Otherwise, code as missing. |
| Weight | Weight (in kg) on the index date or the date closest to the index date during the one-year pre-index period. Retain values that are within 3 SD of the mean of all weights for the cohort. For Veterans without observations within 3 SD of the mean, other values will be accepted as valid if there are at least 2 observations within 2.27 kilograms. Otherwise, code as missing. |
| Body mass index | Body mass index on or closest to the index date during the one-year pre-index period. Height and weight measurements do not need to be on the same day. This variable will be calculated from height and weight observations as weight (in kilograms) divided by height (in meters, squared). |
| Systolic blood pressure | Most recent proximal SBP value corresponding to an outpatient encounter in cardiology, renal, and primary care settings in the one-year pre-index period (including the index date). SBP values will be dropped if any of the following was true: missing value (either SBP or DBP), systolic less than diastolic, systolic >300 mmHg, or systolic <60 mmHg. |
| Diastolic blood pressure | Most recent proximal DBP value corresponding to an outpatient encounter in cardiology, renal, and primary care settings in the one-year pre-index period (including the index date). DBP values will be dropped if any of the following was true: missing value (either SBP or DBP), diastolic greater than systolic, diastolic <30 mmHg, or diastolic >180 mmHg. |
| Heart rate | Most recent proximal heart rate/pulse value corresponding to an outpatient encounter in the one-year pre-index period (including the index date). |
| Total cholesterol | The total cholesterol value closest to the index date in the one-year pre-index period. Defined using OMOP’s mapping where LOINC is '2093-3'. |
| HDL-C level | The HDL-C value closest to the index date in the one-year pre-index period. Defined using OMOP’s mapping where LOINC is '2085-9'. |
| LDL-C level | The LDL-C value closest to the index date in the one-year pre-index period. Defined using OMOP’s mapping where LOINC is '13457-7', '18262-6', '2089-1', '2574-2', or '9346-8'. |
| Triglyceride level | The triglyceride value closest to the index date in the one-year pre-index period. Defined using OMOP’s mapping where LOINC is '2571-8'. |
| Hemoglobin A1c | The glycated hemoglobin (i.e., “hemoglobin A1c”) value closest to the index date in the one-year pre-index period. Defined using OMOP’s mapping where LOINC is '4548-4'. |
| Serum creatinine | The serum creatinine value closest to the index date in the one-year pre-index period. Defined using OMOP’s mapping where LOINC is '2160-0'. |
| Estimated glomerular filtration rate | The estimated glomerular filtration rate closest to the index date in the one-year pre-index period. The VA calculates eGFR using the Modified Diet in Renal Disease equation.(7) |
| Serum potassium | The serum potassium value closest to the index date in the one-year pre-index period. Defined using OMOP’s mapping where LOINC is '6298-4' or '2823-3'. |
| Diabetes | Any of the following using all available claims prior to the index date (including the index date):   1. ICD-9 codes:    1. ≥1 inpatient claim with a discharge diagnosis code (any position) of 250.xx, 357.2, 362.0x, or 366.41.    2. At least 2 outpatient claims with diagnosis code (any position) of 250.xx, 357.2, 362.0x, or 366.41, with the 2 claims occurring at least 7 days apart. 2. ICD-10 codes:    1. ≥1 inpatient claim with a discharge diagnosis code (any position) of E0836, E08.42, E09.36, E09.42, E10.10, E10.11, E10.29, E10.311, E10.319, E10.36, E10.39, E10.40, E10.42, E10.51, E10.618, E10.620, E10.621, E10.622, E10.628, E10.630, E10.638, E10.641, E10.649, E10.65, E10.69, E10.8, E10.9, E11.00, E11.01, E11.29, E11.311, E11.319, E11.329, E11.339, E11.349, E11.359, E11.36, E11.39, E11.40, E11.42, E11.51, E11.618, E11.620, E11.621, E11.622, E11.628, E11.630, E11.638, E11.641, E11.649, E11.65, E11.69, E11.8, E11.9, E13.10, E13.36, E13.42.    2. At least 2 outpatient claims with diagnosis code (any position) of E0836, E08.42, E09.36, E09.42, E10.10, E10.11, E10.29, E10.311, E10.319, E10.36, E10.39, E10.40, E10.42, E10.51, E10.618, E10.620, E10.621, E10.622, E10.628, E10.630, E10.638, E10.641, E10.649, E10.65, E10.69, E10.8, E10.9, E11.00, E11.01, E11.29, E11.311, E11.319, E11.329, E11.339, E11.349, E11.359, E11.36, E11.39, E11.40, E11.42, E11.51, E11.618, E11.620, E11.621, E11.622, E11.628, E11.630, E11.638, E11.641, E11.649, E11.65, E11.69, E11.8, E11.9, E13.10, E13.36, E13.42, with the 2 claims occurring at least 7 days apart. 3. ≥1 pharmacy claim for an oral antidiabetic drug fill or insulin in the one-year pre-index period. |
| Chronic kidney disease(8, 9) | Any of the following using all available claims prior to the index date (including the index date):   1. ICD-9 codes:    1. ≥1 inpatient claim with a discharge diagnosis code (any position) of 250.xx, 357.2, 362.0x, or 366.41.    2. At least 2 outpatient claims with diagnosis code (any position) of 250.xx, 357.2, 362.0x, or 366.41, with the 2 claims occurring at least 7 days apart. 2. ICD-10 codes:    1. ≥1 inpatient claim with a discharge diagnosis code (any position) of A18.11, A52.75, C6.49, C6.89, D30.00, D41.00, D41.20, D59.3, E10.21,E10.29, E11.21, E11.29, E74.8, I12.0, I129, I13.0, I13.10, I13.11, I13.2, I70.1, I72.2, K76.7, M10.30, N00.3, N00.8, N00.9, N01.3, N02.2, N03.2, N03.3, N03.5, N03.8, N03.9, N04.0, N04.3, N04.4, N04.8, N04.9, N05.2, N05.5, N05.8, N05.9, N08.x, N13.30, N17.0, N17.1, N17.2, N17.8, N17.9, N18.1, N18.2, N18.3, N18.4, N18.5, N18.6, N18.9, N19.x, N25.0, N25.1, N25.81, N25.89, N25.9, N26.9,Q61.02, Q61.19, Q61.2, Q61.3, Q61.4, Q61.5, Q61.8, Q62.10, Q62.11, Q62.12, Q62.31, Q62.39, R94.4) .    2. ≥1 physician evaluation and management visit with a diagnosis code (any position) of A18.11, A52.75, C6.49, C6.89, D30.00, D41.00, D41.20, D59.3, E10.21,E10.29, E11.21, E11.29, E74.8, I12.0, I129, I13.0, I13.10, I13.11, I13.2, I70.1, I72.2, K76.7, M10.30, N00.3, N00.8, N00.9, N01.3, N02.2, N03.2, N03.3, N03.5, N03.8, N03.9, N04.0, N04.3, N04.4, N04.8, N04.9, N05.2, N05.5, N05.8, N05.9, N08.x, N13.30, N17.0, N17.1, N17.2, N17.8, N17.9, N18.1, N18.2, N18.3, N18.4, N18.5, N18.6, N18.9, N19.x, N25.0, N25.1, N25.81, N25.89, N25.9, N26.9,Q61.02, Q61.19, Q61.2, Q61.3, Q61.4, Q61.5, Q61.8, Q62.10, Q62.11, Q62.12, Q62.31, Q62.39, R94.4) in any position.    3. Estimated glomerular filtration rate of <60 mL/min/1.73 m^2^. The eGFR value used is the most recent within the one-year pre-index period. To account for data/reading errors, only eGFR values between 0 and 250 will be considered. |
| Heart failure with reduced ejection fraction | Defined as left ventricular ejection fraction (LVEF) value ≤40% in the one-year pre-index period. If there are multiple measurements on one date or if a range is provided, we will take the average of the two measurements. If no LVEF measurement is available, then we will look for the presence of one of the following using all available claims before the index date:   1. ICD-9 codes:    1. ≥1 inpatient claim with discharge diagnosis code (any position) of 428.0x, 428.1x, 428.2x, or 428.4x.    2. ≥2 outpatient or carrier claims on separate calendar days with diagnosis code (any position) of 428.0x, 428.1x, 428.2x, or 428.4x. 2. ICD-10 codes:    1. ≥1 inpatient claim with discharge diagnosis code (any position) of I50.1, I50.2x, I50.4x, or I50.9.    2. ≥2 outpatient or carrier claims on separate calendar days with diagnosis code (any position) of I50.1, I50.2x, I50.4x, or I50.9.    3. At least one prescription for sacubitril/valsartan in the 104 days prior to the index date. |
| History of CHD(10) | Any of the following using all available claims prior to the index date (including the index date):   1. ICD-9 codes:    1. ≥1 inpatient claim with a discharge diagnosis code (any position) of 410.xx-414.xx, V45.81, or V45.82.    2. At least 2 carrier claims, carrier line, or outpatient claims with diagnosis code (any position) of 410.xx-414.xx, V45.81, or V45.82. 2. ICD-10 codes:    1. ≥1 inpatient claim with a discharge diagnosis code (any position) of I20.0, I21.xx, I22.xx, I24.0, I24.8, I24.9, I25.10, I25.110, I25.700, I25.710, I25.720, I25.730, I25.750, I25.760, I25.790, I25.810, I25.811, I25.812, I25.3, I25.41, I25.42, Z95.1, or Z9861.    2. At least 2 outpatient or carrier claims, with diagnosis codes of codes I20.0, I21.xx, I22.xx, I24.0, I24.8, I24.9, I25.10, I25.110, I25.700, I25.710, I25.720, I25.730, I25.750, I25.760, I25.790, I25.810, I25.811, I25.812, I25.3, I25.41, I25.42, Z95.1, or Z98.61. 3. Veterans who met the definition of a prior coronary revascularization, as defined below, will be also considered to have a history of CHD. |
| Prior coronary revascularization | Defined by ≥1 inpatient or outpatient procedure with a CPT code for coronary revascularization (33510-33519, 33521-33523, 33530, 33533-33536, 92920, 92921, 92924, 92925, 92928, 92929, 92933, 92934, 92937, 92938, 92941, 92943, 92944, 92980, 92981, 92982, 92984, or 92996), an ICD-9 procedure code (any position) of 00.66, 36.0, 36.01-36.19, or 36.2, or an ICD-10 procedure code starting with any of the following: 0210, 0211, 0212, 0213, 0270, 0271, 0272, 0273, 02C0, 02C1, 02C2, 02C3, or 3E07 using all available claims prior to the index date (including the index date). In addition to having 1 inpatient or outpatient procedure, Veterans are required to meet ≥1of the following criteria:   1. Have no inpatient claims with a discharge diagnosis code for acute myocardial infarction (ICD-9 codes 410.x0 or 410.x1 or ICD-10 codes I21.xx or I22.xx) within 60 days prior to the procedure. 2. Have primary discharge diagnosis codes for non-elective CHD-related hospitalization prior to the index date (including the index date): 3. Arrhythmia: ICD-9 diagnosis code of 427.xx [except 427.5] or ICD-10 diagnosis code of I47.1, I47.2, I47.9, I48.91, I48.92, I49.01, I49.02, I49.1, I49.3, I49.40, I49.49, I49.5, I49.8, I49.9, R00.1. 4. Cardiac arrest: ICD-9 diagnosis code of 427.5, or ICD-10 diagnosis code of I46.9. 5. Heart failure: ICD-9 diagnosis code of 402.01, 402.11, 402.91, 404.01, 404.03, 404.11, 404.13, 404.91, 404.93, or 428.x, or ICD-10 diagnosis code of I11.0, I13.0, I13.2, I50.1, I50.20, I50.21, I50.22, I50.23, I50.30, I50.31, I50.32, I50.33, I50.40, I50.41, I50.42, I50.43, or I50.9. 6. Unstable angina: ICD-9 diagnosis code of 411.xx or ICD-10 diagnosis code of I20.0, I24.0, I24.1, I24.8. |
| History of Stroke(11) | Any of the following using all available claims prior to the index date (including the index date):   1. ICD-9 codes:    1. ≥1 inpatient claim with a discharge diagnosis code (primary or secondary position) of 433.x1 or 434.x1.    2. ≥1 outpatient claim with a diagnosis code (any position) of 433.x1 or 434.x1. 2. ICD-10 codes:    1. ≥1 inpatient discharge diagnosis code (primary or secondary position) of I63.xx.    2. ≥1 outpatient claim with diagnosis code (any position) of I63.xx.    3. ≥1 inpatient ICD-10 procedure code of 03CH0ZZ, 03CH4ZZ, 03CJ0ZZ, 03CJ4ZZ, 03CK0ZZ, 03CK4ZZ, 03CL0ZZ, 03CL4ZZ, 03CM0ZZ, 03CM4ZZ, 03CN0ZZ, 03CN4ZZ, 03RH07Z, 03RH0JZ, 03RH0KZ, 03RH47Z, 03RH4JZ, 03RH4KZ, 03RJ07Z, 03RJ0JZ, 03RJ0KZ, 03RJ47Z, 03RJ4JZ, 03RJ4KZ,03RK07Z, 03RK0JZ, 03RK0KZ, 03RK47Z, 03RK4JZ, 03RK4KZ, 03RL07Z, 03RL0JZ, 03RL0KZ, 03RL47Z, 03RL4JZ, 03RL4KZ, 03RM07Z, 03RM0JZ, 03RM0KZ, 03RM47Z, 03RM4JZ, 03RM4KZ, 03RN07Z, 03RN0JZ, 03RN0KZ, 03RN47Z, 03RN4JZ, or 03RN4KZ. 3. CPT codes:    1. ≥1 inpatient or outpatient claim with a CPT code for carotid revascularization of 35301, 35390, 37215, 37216, 0005T, 0075T, or 0076. |
| History of PAD | Any of the following using all available claims prior to the index date (including the index date):   1. ICD-9 codes:    1. ≥1 inpatient claim with a discharge diagnosis code (any position) of 440.20-440.24, 440.31, 444.2, 443.9, or 444.81.    2. ≥2 physician evaluation and management outpatient or carrier claims with diagnosis code (any position) of 440.20-440.24, 440.31, 444.2, 443.9, or 444.81 on separate days. 2. ICD-10 codes:    1. ≥1 inpatient claim with a discharge diagnosis code (any position) of I70.209, I70.219, I70.229, I70.25, I70.269, I70.499, I73.9.    2. ≥2 physician evaluation and management outpatient or carrier claims with a diagnosis code (any position) of I70.209, I70.219, I70.229, I70.25, I70.269, I70.499, I73.9 on separate days. 3. CPT code: ≥1 inpatient, outpatient, or carrier claim with a CPT code of 37205 or 75962. |
| History of ASCVD | Defined by a history of CHD, cerebrovascular disease, or peripheral artery disease, as defined above. |
| Kidney failure | Any of the following using all available claims prior to the index date (including the index date):   1. ICD-9 codes:    1. ≥1 inpatient claim with a discharge diagnosis code (any position) of 585.5.    2. ≥2 physician evaluation and management outpatient or carrier claims with a diagnosis code (any position) of 585.5. 2. ICD-10 codes:    1. ≥1 inpatient claim with a discharge diagnosis code (any position) of N18.6.   ≥2 physician evaluation and management outpatient or carrier claims with a diagnosis code (any position) of N18.6. |
| History of kidney transplant | Any of the following using all available claims prior to the index date (including the index date):   1. ICD-9 codes:    1. ≥1 inpatient claim with a discharge diagnosis code (any position) of V42.0.    2. ≥2 physician evaluation and management outpatient or carrier claims with a diagnosis code (any position) of V42.0. 2. ICD-10 codes:    1. ≥1 inpatient claim with a discharge diagnosis code (any position) of Z94.0.    2. ≥2 physician evaluation and management outpatient or carrier claims with a diagnosis code (any position) of Z94.0. |
| Atrial fibrillation(12) | Any of the following using all available claims prior to the index date (including the index date):   1. ICD-9 codes:    1. ≥1 inpatient claim with a discharge diagnosis code (any position) of 427.31.    2. ≥2 physician evaluation and management outpatient or carrier claims with a diagnosis code (any position) of 427.31. 2. ICD-10 codes:    1. ≥1 inpatient claim with a discharge diagnosis code (any position) of I48.0, I48.2, I48.91.    2. ≥2 physician evaluation and management outpatient or carrier claims with a diagnosis code (any position) of I48.0, I48.2, I48.91. |
| Chronic obstructive pulmonary disease(13) | Any of the following using all available claims prior to the index date (including the index date):   1. ICD-9 codes:    1. ≥1 inpatient claim with a discharge diagnosis code (any position) of 491-492 or 496.    2. ≥2 physician evaluation and management outpatient or carrier claims with a diagnosis code (any position) of 491-492 or 496. 2. ICD-10 codes:    1. ≥1 inpatient claim with a discharge diagnosis code (any position) of J41-J44.    2. ≥2 physician evaluation and management outpatient or carrier claims with a diagnosis code (any position) of J41-J44. |
| Asthma(14) | Any of the following using all available claims prior to the index date (including the index date):   1. ICD-9 codes:    1. ≥1 inpatient claim with a discharge diagnosis code (any position) of 493.    2. ≥2 physician evaluation and management outpatient or carrier claims with a diagnosis code (any position) of 493. 2. ICD-10 codes:    1. ≥1 inpatient claim with a discharge diagnosis code (any position) of J45.    2. ≥2 physician evaluation and management outpatient or carrier claims with a diagnosis code (any position) of J45. |
| History of depression(15) | Any of the following using all available claims prior to the index date (including the index date):   1. ICD-9 codes:    1. ≥1 inpatient claim with a discharge diagnosis code (any position) of 296.2, 296.3, 296.5, 300.4, 309.x, or 311.    2. ≥2 physician evaluation and management outpatient or carrier claims with a diagnosis code (any position) of 296.2, 296.3, 296.5, 300.4, 309.x, or 311. 2. ICD-10 codes:    1. ≥1 inpatient claim with a discharge diagnosis code (any position) of F20.4, F31.3-F31.5, F32.x, F33.x, F34.1, F41.2, or F43.2.    2. ≥2 physician evaluation and management outpatient or carrier claims with a diagnosis code (any position) of F20.4, F31.3-F31.5, F32.x, F33.x, F34.1, F41.2, or F43.2. |
| Charlson Comorbidity Index(16) | Continuous variable to represent chronic disease burden. Calculated according to methods described in Quan et al (incorporates Elixhauser and Deyo comorbidities). |
| Current statin use | Defined as one or more pharmacy fills for a statin in the 104 days prior to each Veteran’s index date (atorvastatin, rosuvastatin, simvastatin, fluvastatin, pitavastatin, pravastatin, or lovastatin). |
| Current aspirin use | Defined as one or more pharmacy fills for aspirin in the 104 days prior to each Veteran’s index date. |
|  |  |
| *Exposures* |  |
| Non-ARB/ACEI-based antihypertensive regimen | Defined as one or more pharmacy fills for an oral non-ARB or ACEI medication in the 90 days (+ 14 days) prior to each Veterans index date without a fill for an ACEI or an ARB medication in the same 90+14 day pre-index period. Categorized by class as aldosterone receptor antagonist, alpha blocker, beta blocker, calcium channel blocker, centrally-acting drug, direct arterial vasodilator, direct renin inhibitor, thiazide diuretic, loop diuretic, and potassium-sparing diuretic. See Supplemental Table S2 for specific drug names within each class. Excluded non-oral products, except for clonidine patches. |
| ARB/ACEI-based antihypertensive regimen | Defined as one or more pharmacy fills for an oral ACEI or an ARB in the 90 days (+14 days) prior to each Veteran’s index date. See Supplemental Table S2 for specific drug names within each class. Excluded non-oral products. Exclude sacubitril/valsartan (Brand name: Entresto®) from ARB exposures. |
| ACEI | Defined as one or more pharmacy fills for an oral ACEI in the 90 days (+14 days) prior to each Veteran’s index date. See Supplemental Table S2 for specific drug names within each class. Excluded non-oral products. |
| ARB | Defined as one or more pharmacy fills for an oral ARB in the 90 days (+14 days) prior to each Veteran’s index date. See Supplemental Table S2 for specific drug names within each class. Excluded non-oral products. Exclude sacubitril/valsartan (Brand name: Entresto®) from ARB exposures. |
| Antihypertensive medication daily dose | Calculated for each antihypertensive medication being taken as milligrams of drug taken per day. We will first parse the dosage value then use the quantity and days supplied variables to inform the parsed dose value (if partial or multiple pills are being taken per day, for example). |
| Number of antihypertensive medication fills | Calculated for each antihypertensive medication being taken. Number of antihypertensive medication fills in the most proximal 12 months prior each Veteran’s index date. Used only in a sensitivity analysis. |
| Alternative exposure definition 1 | Used for a sensitivity analysis. Described as a dichotomous variable (yes=1; no=0). Classified as “yes” if the patient had ≥1 pharmacy fills for the primary exposure of interest in each of the two, six-month periods during the 180 days (+14 days) prior to the index date (including the index date). The pharmacy fills do not have to be consecutive. |
| Alternative exposure definition 2 | Used for a sensitivity analysis. Described as a dichotomous variable (yes=1; no=0). Classified as “yes” if the patient had ≥1 pharmacy fill in the 90 days (+14 days) prior to the index date (including the index date) for the primary exposure of interest and 3 consecutive pharmacy fills for the primary exposure of interest in the 1 year (+ 14 days) prior to the index date (including the index date). A “consecutive” fill is defined as pharmacy fills with less than 30 days of gap in days’ supply between the fills. The patient must meet the definition of the exposure group for each fill, regardless of medication class (e.g., if the patient filled thiazide, CCB, then loop diuretic, patient would meet criteria to be included in this cohort, even though it wasn’t the same drug class filled each time). |
| Alternative exposure definition 3 | Used for a sensitivity analysis. Described as a dichotomous variable (yes=1; no=0). Classified as “yes” if a prescription for the primary exposure of interest was dispensed before the index date with a day supply that met or exceeded the index date. Classified as “no” if prescription was dispensed before the index date with a day supply that did not meet or exceed the index date. |
| Alternative exposure definition 4 (inpatient only) | Used for a sensitivity analysis. Described as a dichotomous variable (yes=1; no=0). Classified as “yes” if the patient had 1 pharmacy fill in the 90 days prior to the index date and at least 1 medication administration for the same antihypertensive medication after the index date (including the index date). |
|  |  |
| *Outcomes* |  |
| Time to event | Integer number of days from index date to each outcome below post the index date, separately. If event occurs on index date, time to event = 0.5. |
| All-cause hospitalization | A binary variable indicating the occurrence of 1 or more all-cause hospitalization(s) during the post-index period. |
| All-cause mortality | A dichotomous variable indicating all-cause mortality during the post-index period. |
| ICU admission | A dichotomous variable indicating admission to an intensive care unit during the first hospitalization post-index date. |
| Dialysis | A dichotomous variable indicating occurrence of dialysis post-index date, inclusive of admit and discharge dates. Veterans with an ICD-9 or ICD-10 code for Kidney failure (N18.6 or 585.5) at any time prior to the index date were excluded prior to calculation of this outcome. Occurrence of any of the following post-index date (including the index date) will indicate dialysis:   1. Stop codes 602, 603, 604, 605, 606, 607, 609, 610, and 611, OR 2. Current Procedural Terminology: 90935, 90937, 90945, 90947, 90999, OR   ICD-10 procedure code: Z49.x |
| Mechanical ventilation | Occurrence of any of the following codes associated with a hospital (inpatient) encounter post-index date (including the index date):   1. ICD-10 codes: J95.850, Z99.1x, or T88.4x 2. Current Procedural Terminology: 31500 3. ICD-10 procedure codes: 5A19054, 5A0945Z, 5A1935Z, 5A1955Z, or 5A0935Z 4. Order for ventilation placed in the electronic health record 5. Inpatient medication administration of a paralytic agent (succinylcholine, cisatracurium, or rocuronium) |
| Gastrointestinal bleeding(17) (negative control outcome) | Occurrence of any of the following codes associated with a hospital (inpatient) encounter post-index date (including the index date):   1. ICD-10 code in any position for K25-K28 or K92.2. 2. CPT code 78278, 7424.x, 7425.x, 74260, 435.xx, 436.xx, 440.xx, 44120, or 446.xx |
| Urinary tract infection | Presence of a positive urine culture, defined as urine culture with at least one organism with a quantitative count of >10^5^ CFU/mL of any of the following uropathogens: Escherichia coli, Pseudomonas aeruginosa, Enterococcus spp., Klebsiella spp., Enterobacter spp., Proteus spp., Citrobacter spp., Providentia spp., Morganella spp., Serratia spp., or Candida spp. during the hospitalization post-index date (including the index date) OR  Presence of the ICD-10 codes N10, N30, N39, or A41.9 using all available claims post the index date. |

| Supplemental Table S2: List of antihypertensive medications by class. | |
| --- | --- |
| **Medication class** | **Medication name** |
| Angiotensin-converting enzyme inhibitor | benazepril |
|  | captopril |
|  | enalapril |
|  | fosinopril |
|  | lisinopril |
|  | moexipril |
|  | perindopril |
|  | quinapril |
|  | ramipril |
|  | trandolapril |
| Angiotensin II receptor blocker | azilsartan |
|  | candesartan |
|  | eprosartan |
|  | irbesartan |
|  | losartan |
|  | olmesartan |
|  | telmisartan |
|  | valsartan* |
| Alpha blocker | doxazosin |
|  | prazosin |
|  | terazosin |
| Beta blocker | acebutolol |
|  | nebivolol |
|  | atenolol |
|  | betaxolol |
|  | bisoprolol |
|  | metoprolol |
|  | pindolol |
|  | penbutolol |
|  | carvedilol |
|  | labetalol |
|  | nadolol |
|  | propranolol |
|  | timolol |
| Calcium channel blocker | amlodipine |
|  | felodipine |
|  | isradipine |
|  | nicardipine |
|  | nifedipine |
|  | nisoldipine |
|  | diltiazem |
|  | verapamil |
| Centrally acting | clonidine |
|  | guanabenz |
|  | guanfacine |
|  | methyldopa |
|  | reserpine |
| Direct vasodilators | hydralazine |
|  | minoxidil |
| Direct renin inhibitor | aliskiren |
| Aldosterone receptor antagonist | spironolactone |
|  | eplerenone |
| Loop diuretic | bumetanide |
|  | ethacrynic acid |
|  | furosemide |
|  | torsemide |
| Potassium-sparing diuretic | amiloride |
|  | triamterene |
| Thiazide diuretic | bendroflumethiazide |
|  | chlorothiazide |
|  | chlorthalidone |
|  | hydrochlorothiazide |
|  | indapamide |
|  | metolazone |
| *Excluding sacubitril/valsartan products. | |

| **Supplemental Table S3: Covariates included in the estimation of the propensity scores by exposure group.** | | |
| --- | --- | --- |
| Characteristic | ARB/ACEI-based antihypertensive regimen vs. non-ARB/ACEI-based antihypertensive regimen comparison | ARB user vs. ACEI user comparison |
| Index month | X | X |
| VISN | X | X |
| Age | X | X |
| Sex | X | X |
| Race-ethnicity | X | X |
| Income | X | X |
| Insurance type | X | X |
| Priority group status | X | X |
| Current tobacco use | X | X |
| Body mass index | X | X |
| Systolic blood pressure | X | X |
| Diastolic blood pressure | X | X |
| Heart rate | X | X |
| Total cholesterol | X | X |
| High-density lipoprotein cholesterol | X | X |
| Low-density lipoprotein cholesterol | X | X |
| Triglycerides | X | X |
| Hemoglobin A1c | X | X |
| Serum potassium | X | X |
| Serum creatinine | X | X |
| Estimated glomerular filtration rate | X | X |
| Diabetes | n/a | X |
| Chronic kidney disease | n/a | X |
| Heart failure with reduced ejection fraction | n/a | X |
| Coronary heart disease | n/a | X |
| History of stroke | n/a | X |
| Peripheral artery disease | X | X |
| Atrial fibrillation | X | X |
| Chronic obstructive pulmonary disease | X | X |
| Asthma | X | X |
| Depression | X | X |
| Charlson Comorbidity Index | X | X |
| Calcium channel blocker | * | X |
| Thiazide diuretic | * | X |
| Alpha blocker | * | X |
| Beta blocker | * | X |
| Centrally-acting agent | * | X |
| Direct arterial vasodilator | * | X |
| Direct renin inhibitor | * | X |
| Aldosterone receptor antagonist | * | X |
| Loop diuretic | * | X |
| Potassium-sparing diuretic | * | X |
| Number of antihypertensive medications in regimen | * | X |
| Statin use | X | X |
| Aspirin use | X | X |
| An X indicates that the variable was included in the estimation of the propensity score. | | |
| *Variables not included in calculation of propensity score for the comparison of ARB/ACEI-based antihypertensive regimen vs. non-ARB/ACEI-based antihypertensive regimen comparison because these variables are constructs of the main exposure. | | |
| ACEI: angiotensin-converting enzyme inhibitor; ARB: angiotensin II receptor blocker; VISN: Veterans Integrated Service Network | | |

| Supplemental Table S4: Baseline characteristics of outpatient Veterans with treated hypertension without compelling indications for an ARB or ACEI who are current users of an ARB/ACEI-based antihypertensive regimen vs. non-ARB/ACEI-based antihypertensive regimen, before and after propensity score weighting. | | | | | | | |
| --- | --- | --- | --- | --- | --- | --- | --- |
| **Patient characteristics** | **% missing** | **Before weighting** | | | **After weighting** | | |
|  |  | **ARB/ACEI-based antihypertensive regimen** | **Non-ARB/ACEI-based antihypertensive regimen** | **ASMD** | **ARB/ACEI-based antihypertensive regimen** | **Non-ARB/ACEI-based antihypertensive regimen** | **ASMD** |
|  |  | **(n = 2,482)** | **(n = 2,487)** |  | **(n = 2,482)** | **(n = 2,487)** |  |
| ***Demographics*** |  |  |  |  |  |  |  |
| Age (years) | 0.0 | 60.3 (12.6) | 59.7 (13.2) | 0.0465 | 60.1 (12.8) | 60.1 (13.0) | 0.0014 |
| Male sex | 0.0 | 2277 (91.7) | 2115 (85.0) | 0.2433 | 90.0 | 90.1 | 0.0043 |
| Race-ethnicity | 5.9 |  |  |  |  |  |  |
| Non-Hispanic White |  | 1270 (51.2) | 1053 (42.3) | 0.1766 | 47.0 | 46.9 | 0.0024 |
| Non-Hispanic Black |  | 788 (31.8) | 1062 (42.7) | 0.2353 | 36.7 | 36.9 | 0.0042 |
| Hispanic |  | 253 (10.2) | 217 (8.7) | 0.0485 | 9.7 | 9.7 | 0.0003 |
| Asian American |  | 19 (0.8) | 13 (0.5) | 0.0279 | 0.7 | 0.6 | 0.0032 |
| Other/Missing |  | 152 (6.1) | 142 (5.7) | 0.0173 | 6.0 | 5.9 | 0.0022 |
| Median area-level income, USD | 8.3 | 56,452 (14,920) | 56,755 (14,228) | 0.0203 | 56,469 (14,940) | 56,521 (14,056) | 0.0035 |
| Commercial health insurance | 0.0 | 570 (23.0) | 486 (19.5) | 0.0814 | 20.9 | 21.0 | 0.0020 |
| Priority group status 1 | 0.0 | 1172 (47.2) | 1247 (50.1) | 0.0585 | 48.7 | 48.8 | 0.0025 |
| Current tobacco use | 0.0 | 274 (11.0) | 314 (12.6) | 0.0506 | 11.5 | 11.7 | 0.0049 |
| ***Vitals and laboratory measurements*** |  |  |  |  |  |  |  |
| Body mass index, kg/m^2^ | 31.0 | 32.1 (5.1) | 31.4 (5.1) | 0.1453 | 31.8 (4.8) | 31.8 (5.1) | 0.0003 |
| Systolic BP, mm Hg | 11.2 | 132.1 (13.9) | 131.3 (13.3) | 0.0595 | 131.7 (13.6) | 131.7 (13.4) | 0.0027 |
| Diastolic BP, mm Hg | 11.2 | 80.1 (9.2) | 79.7 (8.9) | 0.0432 | 79.9 (9.1) | 79.9 (9.0) | 0.0024 |
| Total cholesterol, mg/dL | 23.7 | 175.2 (36.3) | 176.3 (35.4) | 0.0292 | 176.0 (36.1) | 176.0 (35.6) | 0.0012 |
| LDL-C, mg/dL | 29.5 | 105.8 (29.5) | 106.7 (29.0) | 0.0285 | 106.4 (29.2) | 106.4 (29.6) | 0.0034 |
| HDL-C, mg/dL | 23.9 | 48.4 (15.1) | 49.8 (15.3) | 0.0924 | 49.1 (15.0) | 49.1 (15.3) | 0.0001 |
| Triglycerides, mg/dL | 25.0 | 143.6 (81.7) | 141.0 (84.6) | 0.0320 | 143.2 (81.9) | 143.3 (86.6) | 0.0016 |
| Hemoglobin A1c, % | 57.2 | 5.3 (0.5) | 5.3 (0.4) | 0.0877 | 5.3 (0.4) | 5.3 (0.4) | 0.0010 |
| Serum potassium, mEq/L | 11.6 | 4.1 (0.4) | 4.1 (0.4) | 0.1401 | 4.1 (0.4) | 4.1 (0.4) | 0.0092 |
| Serum creatinine, mg/dL | 10.4 | 1.0 (0.2) | 1.0 (0.2) | 0.0680 | 1.0 (0.2) | 1.0 (0.2) | 0.0008 |
| eGFR, mL/min/1.73 m^2^ | 31.9 | 85.5 (14.1) | 86.9 (15.1) | 0.0988 | 86.2 (14.3) | 86.2 (14.8) | 0.0008 |
| ***Comorbidities*** |  |  |  |  |  |  |  |
| Peripheral artery disease | 0.0 | 66 (2.7) | 67 (2.7) | 0.0022 | 2.6 | 2.6 | 0.0020 |
| Kidney failure | 0.0 | 0 (0.0) | 0 (0.0) | 0.0000 | 0.0 | 0.0 | 0.0000 |
| History of kidney transplant | 0.0 | 0 (0.0) | 0 (0.0) | 0.0000 | 0.0 | 0.0 | 0.0000 |
| Atrial fibrillation | 0.0 | 102 (4.1) | 153 (6.2) | 0.1029 | 4.9 | 4.9 |  |
| Chronic obstructive pulmonary disease | 0.0 | 287 (11.6) | 310 (12.5) | 0.0282 | 12.0 | 12.0 | 0.0005 |
| Asthma | 0.0 | 195 (1.9) | 253 (10.2) | 0.0861 | 8.8 | 8.7 | 0.0023 |
| Depression | 0.0 | 1249 (50.3) | 1426 (57.3) | 0.1403 | 53.4 | 53.4 | 0.0050 |
| Charlson Comorbidity Index, mean (SD) | 0.0 | 0.7 (1.3) | 0.8 (1.5) | 0.0946 | 0.7 (1.3) | 0.7 (1.4) | 0.0012 |
| ***Antihypertensive medication**** |  |  |  |  |  |  |  |
| ACEI | 0.0 | 1673 (67.4) | 0 (0.0) | n/a | 67.2 | 0.0 | n/a |
| ARB | 0.0 | 819 (33.0) | 0 (0.0) | n/a | 33.3 | 0.0 | n/a |
| CCB | 0.0 | 744 (30.0) | 1266 (50.9) | n/a | 31.1 | 50.7 | n/a |
| Thiazide diuretic | 0.0 | 976 (39.3) | 757 (30.4) | n/a | 40.0 | 29.9 | n/a |
| Alpha blocker | 0.0 | 202 (8.1) | 416 (16.7) | n/a | 8.2 | 16.7 | n/a |
| Beta blocker | 0.0 | 428 (17.2) | 737 (29.6) | n/a | 17.5 | 30.1 | n/a |
| Centrally acting | 0.0 | 17 (0.7) | 26 (1.1) | n/a | 0.7 | 1.0 | n/a |
| Direct vasodilator | 0.0 | 24 (1.0) | 12 (0.5) | n/a | 1.1 | 0.5 | n/a |
| Direct renin inhibitor | 0.0 | 0 (0.0) | 0 (0.0) | n/a | 0.0 | 0.0 | n/a |
| Aldosterone receptor antagonist | 0.0 | 29 (1.2) | 47 (1.9) | n/a | 1.1 | 1.9 | n/a |
| Loop diuretic | 0.0 | 61 (2.5) | 82 (3.3) | n/a | 2.4 | 3.3 | n/a |
| Potassium-sparing diuretic | 0.0 | 28 (1.1) | 87 (3.5) | n/a | 1.2 | 3.4 | n/a |
| Total number of antihypertensive medications in regimen | 0.0 | 2.0 (0.9) | 1.4 (0.6) | n/a | 2.0 (0.9) | 1.4 (0.6) | n/a |
| ***Other medication use*** |  |  |  |  |  |  |  |
| Current statin use | 0.0 | 995 (40.1) | 793 (31.9) | 0.1673 | 36.0 | 36.0 | 0.0001 |
| Current aspirin use | 0.0 | 187 (7.5) | 183 (7.4) | 0.0067 | 7.6 | 7.4 | 0.0049 |
| Numbers in table are frequency (column %) or mean (SD) for before-weighting variables, and column % or mean (SD) for after-weighting variables. | | | | | | | |
| ACEI: angiotensin-converting enzyme inhibitor; ARB: angiotensin II receptor blocker; ASMD: absolute standardized mean difference; BP: blood pressure; CCB: calcium channel blocker; eGFR: estimated glomerular filtration rate; HDL-C: high-density lipoprotein cholesterol; LDL-C: low-density lipoprotein cholesterol; SD: standard deviation; USD: United States Dollars | | | | | | | |
| * Variables not included in calculation of propensity score for the comparison of ARB/ACEI-based antihypertensive regimen vs. non-ARB/ACEI-based antihypertensive regimen comparison because these variables are constructs of the main exposure. | | | | | | | |

| Supplemental Table S5: Baseline characteristics of outpatient Veterans with treated hypertension who are current users of an ARB vs. ACEI, before and after propensity score weighting. | | | | | | | |
| --- | --- | --- | --- | --- | --- | --- | --- |
| **Patient characteristics** | **% missing** | **Before weighting** | | | **After weighting** | | |
|  |  | **ARB** | **ACEI** | **ASMD** | **ARB** | **ACEI** | **ASMD** |
|  |  | **(n = 4,877)** | **(n = 8,704)** |  | **(n = 4,877)** | **(n = 8,704)** |  |
| ***Demographics*** |  |  |  |  |  |  |  |
| Age (years) | 0.0 | 66.9 (11.4) | 66.0 (11.7) | 0.0824 | 66.9 (11.4) | 66.9 (11.5) | 0.0026 |
| Male sex | 0.0 | 4540 (93.1) | 8296 (95.3) |  | 93.7 | 93.7 |  |
| Race-ethnicity | 5.4 |  |  |  |  |  |  |
| Non-Hispanic White |  | 2374 (48.7) | 4510 (41.8) | 0.0876 | 49.5 | 49.3 | 0.0003 |
| Non-Hispanic Black |  | 1767 (36.2) | 2731 (31.4) | 0.0628 | 35.3 | 35.4 | 0.0035 |
| Hispanic |  | 455 (9.3) | 925 (10.6) | 0.1010 | 9.4 | 9.5 | 0.0007 |
| Asian American |  | 36 (0.7) | 44 0.5) | 0.0446 | 0.7 | 0.7 | 0.0032 |
| Other/Missing |  | 245 (5.0) | 494 (5.7) | 0.0272 | 5.1 | 5.1 | 0.0017 |
| Median area-level income, USD | 9.0 | 56,241 (14,788) | 55,786 (14,333) | 0.0298 | 56,186 (14,760) | 56,141 (14,564) | 0.0016 |
| Commercial health insurance | 0.0 | 781 (16.0) | 1315 (15.1) | 0.0247 | 15.9 | 15.9 | 0.0027 |
| Priority group status 1 | 0.0 | 2437 (50.0) | 3996 (45.9) | 0.0812 | 49.4 | 49.3 | 0.0019 |
| Current tobacco use | 0.0 | 683 (14.0) | 1304 (15.0) | 0.0282 | 14.1 | 14.0 | 0.0016 |
| ***Vitals and laboratory measurements*** |  |  |  |  |  |  |  |
| Body mass index, kg/m^2^ | 27.3 | 32.5 (5.5) | 32.2 (5.5) | 0.0652 | 32.4 (5.5) | 32.4 (5.8) | 0.0008 |
| Systolic BP, mm Hg | 8.4 | 133.2 (15.8) | 131.0 (15.4) | 0.1394 | 132.9 (15.5) | 132.9 (16.3) | 0.0044 |
| Diastolic BP, mm Hg | 8.4 | 76.7 (10.0) | 76.4 (9.8) | 0.0301 | 76.6 (9.9) | 76.7 (10.1) | 0.0080 |
| Total cholesterol, mg/dL | 17.8 | 158.3 (39.0) | 160.8 (39.2) | 0.0620 | 158.6 (38.9) | 158.6 (38.7) | 0.0003 |
| LDL-C, mg/dL | 23.1 | 90.6 (31.8) | 93.3 (31.9) | 0.0842 | 90.9 (31.7) | 90.9 (31.3) | 0.0002 |
| HDL-C, mg/dL | 18.0 | 45.0 (13.4) | 45.0 (15.2) | 0.0009 | 45.1 (13.3) | 45.1 (15.0) | 0.0007 |
| Triglycerides, mg/dL | 18.9 | 152.6 (128.7) | 154.6 (105.7) | 0.0154 | 152.6 (129.4) | 152.8 (104.8) | 0.0014 |
| Hemoglobin A1c, % | 43.5 | 6.2 (1.6) | 6.1 (1.6) | 0.0189 | 6.2 (1.6) | 6.2 (1.6) | 0.0001 |
| Serum potassium, mEq/L | 6.1 | 4.1 (0.5) | 4.2 (0.5) | 0.1386 | 4.1 (0.5) | 4.1 (0.5) | 0.0024 |
| Serum creatinine, mg/dL | 4.9 | 1.4 (1.2) | 1.2 (0.8) | 0.1247 | 1.3 (1.0) | 1.3 (1.0) | 0.0034 |
| eGFR, mL/min/1.73 m^2^ | 21.0 | 71.5 (24.7) | 75.0 (23.9) | 0.1396 | 72.3 (24.1) | 72.2 (24.2) | 0.0022 |
| ***Comorbidities*** |  |  |  |  |  |  |  |
| Diabetes | 0.0 | 3059 (62.7) | 5305 (61.0) | 0.0367 | 62.3 | 62.3 | 0.0006 |
| Chronic kidney disease | 0.0 | 3161 (64.8) | 5317 (61.1) | 0.0780 | 64.1 | 64.2 | 0.0002 |
| Heart failure with reduced ejection fraction | 0.0 | 719 (14.7) | 1048 (12.0) | 0.0762 | 14.3 | 14.2 | 0.0025 |
| Coronary heart disease | 0.0 | 1982 (40.6) | 3107 (35.7) | 0.1006 | 40.0 | 40.0 | 0.0003 |
| History of stroke | 0.0 | 429 (8.8) | 790 (9.1) | 0.0099 | 8.8 | 8.8 | 0.0011 |
| Peripheral artery disease | 0.0 | 534 (11.0) | 835 (9.6) | 0.0434 | 10.7 | 10.8 | 0.0022 |
| Kidney failure | 0.0 | 139 (2.9) | 110 (1.3) | 0.0953 | 2.3 | 1.9 | 0.0286 |
| History of kidney transplant | 0.0 | 33 (0.7) | 24 (0.3) | 0.0489 | 0.6 | 0.3 | 0.0374 |
| Atrial fibrillation | 0.0 | 660 (13.5) | 1073 (12.3) | 0.0352 | 13.5 | 13.7 | 0.0062 |
| Chronic obstructive pulmonary disease | 0.0 | 1099 (22.5) | 1759 (20.2) | 0.0556 | 22.2 | 22.2 | 0.0001 |
| Asthma | 0.0 | 539 (11.1) | 739 (8.5) | 0.0817 | 10.5 | 10.4 | 0.0029 |
| Depression | 0.0 | 2525 (51.8) | 4460 (51.2) | 0.0107 | 51.6 | 51.6 | 0.0010 |
| Charlson Comorbidity Index, mean (SD) | 0.0 | 3.0 (2.7) | 2.7 (2.6) | 0.1124 | 2.9 (2.7) | 2.9 (2.7) | 0.0007 |
| ***Antihypertensive medication**** |  |  |  |  |  |  |  |
| ACEI | 0.0 | 0 (0.0) | 8704 (100.0) | n/a | 0.0 | 100.0 | n/a |
| ARB | 0.0 | 4877 (100.0) | 0 (0.0) | n/a | 100.0 | 0.0 | n/a |
| CCB | 0.0 | 1998 (41.0) | 2764 (31.8) | 0.1873 | 39.7 | 40.0 | 0.0054 |
| Thiazide diuretic | 0.0 | 1466 (30.1) | 2632 (30.2) | 0.0039 | 30.3 | 30.4 | 0.0030 |
| Alpha blocker | 0.0 | 459 (9.4) | 734 8.4) | 0.0335 | 9.2 | 9.2 | 0.0021 |
| Beta blocker | 0.0 | 2054 (42.1) | 3194 (36.7) | 0.1098 | 41.4 | 41.6 | 0.0049 |
| Centrally acting | 0.0 | 114 (2.3) | 112 (1.3) | 0.0695 | 2.0 | 2.0 | 0.0014 |
| Direct vasodilator | 0.0 | 257 (5.3) | 233 (2.7) | 0.1160 | 4.3 | 4.3 | 0.0004 |
| Direct renin inhibitor | 0.0 | 0 (0.0) | 0 (0.0) | 0.0000 | 0.0 | 0.0 | 0.0000 |
| Aldosterone receptor antagonist | 0.0 | 282 (5.8) | 311 (3.6) | 0.0946 | 5.2 | 5.2 | 0.0003 |
| Loop diuretic | 0.0 | 185 (16.1) | 1025 (11.8) | 0.1175 | 15.2 | 15.3 | 0.0006 |
| Potassium-sparing diuretic | 0.0 | 74 (1.5) | 83 (1.0) | 0.0461 | 1.4 | 1.4 | 0.0006 |
| Total number of antihypertensive medications in regimen | 0.0 | 2.5 (1.1) | 2.3 (1.1) | 0.2285 | 2.5 (1.1) | 2.5 (1.1) | 0.0052 |
| ***Other medication use*** |  |  |  |  |  |  |  |
| Current statin use | 0.0 | 3147 (64.5) | 5618 (64.6) | 0.0004 | 64.6 | 64.4 | 0.0033 |
| Current aspirin use | 0.0 | 1060 (21.7) | 1841 (21.2) | 0.0141 | 21.6 | 21.5 | 0.0009 |
| Numbers in table are frequency (column %) or mean (SD) for before-weighting variables, and column % or mean (SD) for after-weighting variables. | | | | | | | |
| ACEI: angiotensin-converting enzyme inhibitor; ARB: angiotensin II receptor blocker; ASMD: absolute standardized mean difference; BP: blood pressure; CCB: calcium channel blocker; eGFR: estimated glomerular filtration rate; HDL-C: high-density lipoprotein cholesterol; LDL-C: low-density lipoprotein cholesterol; SD: standard deviation; USD: United States Dollars | | | | | | | |
| * Variables not included in calculation of propensity score because these variables were constructs of the exposure definitions, and therefore will not have ASMD <0.1 after weighting. | | | | | | | |

| Supplemental Table S6: Baseline characteristics of inpatient Veterans with treated hypertension without compelling indications for an ARB or ACEI who are current users of an ARB/ACEI-based antihypertensive regimen vs. non-ARB/ACEI-based antihypertensive regimen, before and after propensity score weighting. | | | | | | | |
| --- | --- | --- | --- | --- | --- | --- | --- |
| **Patient characteristics** | **% missing** | **Before weighting** | | | **After weighting** | | |
|  |  | **ARB/ACEI-based antihypertensive regimen** | **Non-ARB/ACEI-based antihypertensive regimen** | **ASMD** | **ARB/ACEI-based antihypertensive regimen** | **Non-ARB/ACEI-based antihypertensive regimen** | **ASMD** |
|  |  | **(n = 210)** | **(n = 275)** |  | **(n = 210)** | **(n = 275)** |  |
| ***Demographics*** |  |  |  |  |  |  |  |
| Age (years) | 0.0 | 63.5 (12.2) | 62.1 (14.7) | 0.1146 | 63.1 (12.0) | 63.3 (14.4) | 0.0225 |
| Male sex | 0.0 | 197 (93.8) | 240 (87.3) | 0.2706 | 92.0 | 91.7 | 0.0102 |
| Race-ethnicity | 5.2 |  |  |  |  |  |  |
| Non-Hispanic White |  | 93 (44.3) | 121 (44.0) | 0.0057 | 43.4 | 43.8 | 0.0075 |
| Non-Hispanic Black |  | 76 (36.2) | 117 (42.6) | 0.1319 | 39.6 | 39.3 | 0.0062 |
| Hispanic |  | 25 (11.9) | 25 (9.1) | 0.0867 | 11.0 | 10.7 | 0.0116 |
| Asian American |  | 2 (1.0) | 1 (0.4) | 0.0605 | 0.5 | 0.6 | 0.0156 |
| Other/Missing |  | 14 (6.7) | 11 (4.0) | 0.1066 | 5.5 | 5.7 | 0.0085 |
| Median area-level income, USD | 14.4 | 56,850 (13,446) | 58,183 (12,854) | 0.0930 | 56,530 (14,343) | 56,399 (11,372) | 0.0098 |
| Commercial health insurance | 0.0 | 31 (14.8) | 29 (10.6) | 0.1186 | 13.0 | 13.1 | 0.0027 |
| Priority group status 1 | 0.0 | 101 (48.1) | 127 (46.2) | 0.0382 | 47.9 | 47.8 | 0.0008 |
| Current tobacco use | 0.0 | 56 (26.7) | 76 (27.6) | 0.0219 | 25.9 | 25.1 | 0.0179 |
| ***Vitals and laboratory measurements*** |  |  |  |  |  |  |  |
| Body mass index, kg/m^2^ | 19.8 | 32.1 (6.5) | 30.8 (5.5) | 0.1931 | 31.5 (6.7) | 31.3 (5.6) | 0.0280 |
| Systolic BP, mm Hg | 10.5 | 132.5 (14.4) | 131.7 (13.2) | 0.0537 | 132.1 (14.7) | 132.1 (13.6) | 0.0004 |
| Diastolic BP, mm Hg | 10.5 | 78.9 (9.4) | 79.5 (10.1) | 0.0545 | 79.2 (9.5) | 79.1 (10.6) | 0.0096 |
| Total cholesterol, mg/dL | 23.7 | 167.2 (32.9) | 176.1 (37.4) | 0.2715 | 170.0 (32.7) | 169.7 (33.7) | 0.0085 |
| LDL-C, mg/dL | 29.7 | 100.1 (28.0) | 105.8 (31.4) | 0.2032 | 102.6 (28.5) | 102.2 (29.2) | 0.0149 |
| HDL-C, mg/dL | 23.9 | 47.7 (13.2) | 50.1 (15.6) | 0.1636 | 48.1 (14.7) | 48.0 (14.0) | 0.0064 |
| Triglycerides, mg/dL | 24.9 | 139.8 (92.2) | 141.8 (71.5) | 0.0229 | 141.4 (86.4) | 140.6 (67.6) | 0.0085 |
| Hemoglobin A1c, % | 55.7 | 5.3 (0.4) | 5.3 (0.4) | 0.0849 | 5.3 (0.5) | 5.3 (0.4) | 0.0041 |
| Serum potassium, mEq/L | 1.0 | 3.8 (0.4) | 3.8 (0.5) | 0.0769 | 3.8 (0.5) | 3.8 (0.5) | 0.0081 |
| Serum creatinine, mg/dL | 0.0 | 1.0 (0.2) | 1.0 (0.2) | 0.0605 | 1.0 (0.2) | 1.0 (0.2) | 0.0068 |
| eGFR, mL/min/1.73 m^2^ | 23.9 | 87.7 (16.4) | 89.0 (16.5) | 0.1193 | 86.9 (16.3) | 86.7 (15.6) | 0.0113 |
| ***Comorbidities*** |  |  |  |  |  |  |  |
| Peripheral artery disease | 0.0 | 12 (5.7) | 12 (4.4) | 0.0580 | 5.0 | 5.3 | 0.0134 |
| Kidney failure | 0.0 | 0 (0.0) | 0 (0.0) | 0.0000 | 0.0 | 0.0 | 0.0000 |
| History of kidney transplant | 0.0 | 0 (0.0) | 0 (0.0) | 0.0000 | 0.0 | 0.0 | 0.0000 |
| Atrial fibrillation | 0.0 | 19 (9.1) | 23 (8.4) | 0.0238 | 8.5 | 8.0 | 0.0149 |
| Chronic obstructive pulmonary disease | 0.0 | 43 (20.5) | 55 (20.0) | 0.0118 | 19.6 | 19.1 | 0.0116 |
| Asthma | 0.0 | 18 (8.6) | 31 (11.3) | 0.0963 | 8.6 | 9.2 | 0.0209 |
| Depression | 0.0 | 119 (56.7) | 172 (62.6) | 0.1184 | 57.5 | 57.3 | 0.0052 |
| Charlson Comorbidity Index, mean (SD) | 0.0 | 1.0 (1.4) | 1.2 (2.0) | 0.1392 | 1.0 (1.4) | 1.0 (1.7) | 0.0076 |
| ***Antihypertensive medication**** |  |  |  |  |  |  |  |
| ACEI | 0.0 | 148 (70.5) | 0 (0.0) | n/a | 72.1 | 0.0 | n/a |
| ARB | 0.0 | 63 (30.0) | 0 (0.0) | n/a | 28.5 | 0.0 | n/a |
| CCB | 0.0 | 78 (37.1) | 133 (48.4) | n/a | 39.2 | 50.9 | n/a |
| Thiazide diuretic | 0.0 | 84 (40.0) | 90 (32.7) | n/a | 39.8 | 32.3 | n/a |
| Alpha blocker | 0.0 | 24 (11.4) | 47 (17.1) | n/a | 10.1 | 16.7 | n/a |
| Beta blocker | 0.0 | 41 (19.5) | 77 (28.0) | n/a | 18.7 | 27.6 | n/a |
| Centrally acting | 0.0 | 1 (0.5) | 3 (1.1) | n/a | 0.5 | 0.6 | n/a |
| Direct vasodilator | 0.0 | 1 (0.5) | 0 (0.0) | n/a | 0.6 | 0.0 | n/a |
| Direct renin inhibitor | 0.0 | 0 (0.0) | 0 (0.0) | n/a | 0.0 | 0.0 | n/a |
| Aldosterone receptor antagonist | 0.0 | 3 (1.4) | 4 (1.5) | n/a | 1.2 | 0.9 | n/a |
| Loop diuretic | 0.0 | 4 (1.9) | 11 (4.0) | n/a | 1.2 | 4.5 | n/a |
| Potassium-sparing diuretic | 0.0 | 4 (1.9) | 5 (1.8) | n/a | 1.7 | 3.0 | n/a |
| Total number of antihypertensive medications in regimen | 0.0 | 2.1 (0.9) | 1.3 (0.6) | n/a | 2.1 (0.9) | 1. (0.6) | n/a |
| ***Other medication use*** |  |  |  |  |  |  |  |
| Current statin use | 0.0 | 95 (24.2) | 93 (33.8) | 0.2289 | 39.4 | 40.0 | 0.0110 |
| Current aspirin use | 0.0 | 27 (12.9) | 29 (10.6) | 0.0689 | 12.4 | 12.1 | 0.0089 |
| Numbers in table are frequency (column %) or mean (SD) for before-weighting variables, and column % or mean (SD) for after-weighting variables. | | | | | | | |
| ACEI: angiotensin-converting enzyme inhibitor; ARB: angiotensin II receptor blocker; ASMD: absolute standardized mean difference; BP: blood pressure; CCB: calcium channel blocker; eGFR: estimated glomerular filtration rate; HDL-C: high-density lipoprotein cholesterol; LDL-C: low-density lipoprotein cholesterol; SD: standard deviation; USD: United States Dollars | | | | | | | |
| *Variables not included in calculation of propensity score for the comparison of ARB/ACEI-based antihypertensive regimen vs. non-ARB/ACEI-based antihypertensive regimen comparison because these variables are constructs of the main exposure. | | | | | | | |

| Supplemental Table S7: Baseline characteristics of inpatient Veterans with treated hypertension who are current users of an ARB vs. ACEI, before and after propensity score weighting. | | | | | | | |
| --- | --- | --- | --- | --- | --- | --- | --- |
| **Patient characteristics** | **% missing** | **Before weighting** | | | **After weighting** | | |
|  |  | **ARB** | **ACEI** | **ASMD** | **ARB** | **ACEI** | **ASMD** |
|  |  | **(n = 1,164)** | **(n = 2,014)** |  | **(n = 1,164)** | **(n = 2,014)** |  |
| ***Demographics*** |  |  |  |  |  |  |  |
| Age (years) | 0.0 | 69.5 (10.6) | 69.1 (10.7) | 0.0420 | 69.6 (10.5) | 69.6 (10.5) | 0.0048 |
| Male sex | 0.0 | 1103 (94.8) | 1947 (96.7) | 0.0858 |  | 95.4 | 0.0012 |
| Race-ethnicity | 5.0 |  |  |  | 95.5 |  |  |
| Non-Hispanic White |  | 490 (42.1) | 914 (45.4) | 0.0665 | 43.2 | 43.2 | 0.0008 |
| Non-Hispanic Black |  | 474 (40.7) | 788 (39.1) | 0.0325 | 39.8 | 40.0 | 0.0030 |
| Hispanic |  | 124 (10.7) | 207 (10.3) | 0.0121 | 10.6 | 10.5 | 0.0036 |
| Asian American |  | 12 (1.0) | 10 (0.5) | 0.0529 | 0.8 | 0.8 | 0.0013 |
| Other/Missing |  | 64 (5.5) | 95 (4.7) | 0.0343 | 5.5 | 5.5 | 0.0004 |
| Median area-level income, USD | 13.3 | 56,681 (14,573) | 56,203 (13,705) | 0.0328 | 56,536 (14,514) | 56,439 (13,855) | 0.0067 |
| Commercial health insurance | 0.0 | 106 (9.1) | 215 (10.7) | 0.0545 | 9.4 | 9.3 | 0.0004 |
| Priority group status 1 | 0.0 | 526 (45.2) | 828 (41.1) | 0.0819 | 44.7 | 44.7 | 0.0015 |
| Current tobacco use | 0.0 | 340 (29.2) | 617 (30.6) | 0.0313 | 29.0 | 28.9 | 0.0030 |
| ***Vitals and laboratory measurements*** |  |  |  |  |  |  |  |
| Body mass index, kg/m^2^ | 17.5 | 31.8 (6.5) | 31.1 (6.3) | 0.1019 | 31.6 (6.3) | 31.6 (6.5) | 0.0035 |
| Systolic BP, mm Hg | 7.8 | 133.3 (17.1) | 131.7 (16.6) | 0.0963 | 133.1 (16.8) | 133.1 (17.3) | 0.0001 |
| Diastolic BP, mm Hg | 7.8 | 75.2 (10.3) | 75.6 (10.2) | 0.0343 | 75.3 (10.3) | 75.4 (10.4) | 0.0071 |
| Total cholesterol, mg/dL | 19.4 | 153.0 (37.4) | 156.3 (40.3) | 0.0862 | 153.6 (37.5) | 153.7 (38.9) | 0.0043 |
| LDL-C, mg/dL | 25.2 | 87.3 (31.4) | 89.4 (31.5) | 0.0669 | 87.8 (31.4) | 87.9 (30.8) | 0.0021 |
| HDL-C, mg/dL | 19.6 | 45.0 (13.8) | 44.9 (14.0) | 0.0061 | 44.9 (13.0) | 45.1 (14.6) | 0.0110 |
| Triglycerides, mg/dL | 20.3 | 149.3 (100.0) | 150.4 (98.4) | 0.0107 | 149.8 (101.0) | 149.2 (95.3) | 0.0057 |
| Hemoglobin A1c, % | 42.8 | 6.2 (1.7) | 6.3 (1.7) | 0.0221 | 6.2 (1.7) | 6.2 (1.7) | 0.0014 |
| Serum potassium, mEq/L | 1.8 | 4.0 (0.6) | 4.1 (0.5) | 0.0842 | 4.0 (0.6) | 4.0 (0.5) | 0.0001 |
| Serum creatinine, mg/dL | 0.3 | 1.8 (1.7) | 1.5 (1.2) | 0.1771 | 1.7 (1.3) | 1.7 (1.4) | 0.0070 |
| eGFR, mL/min/1.73 m^2^ | 14.8 | 62.0 (29.9) | 67.2 (29.4) | 0.1738 | 63.5 (29.2) | 63.3 (29.6) | 0.0049 |
| ***Comorbidities*** |  |  |  |  |  |  |  |
| Diabetes | 0.0 | 842 (72.3) | 1377 (68.4) | 0.0886 | 71.4 | 71.4 | 0.0006 |
| Chronic kidney disease | 0.0 | 954 (82.0) | 1627 (80.8) | 0.0305 | 81.5 | 81.7 | 0.0049 |
| Heart failure with reduced ejection fraction | 0.0 | 284 (24.4) | 425 (21.1) | 0.0767 | 23.9 | 23.9 | 0.0006 |
| Coronary heart disease | 0.0 | 607 (42.2) | 937 (46.5) | 0.1125 | 51.3 | 51.2 | 0.0014 |
| History of stroke | 0.0 | 170 (14.6) | 301 (15.0) | 0.0096 | 14.5 | 14.3 | 0.0066 |
| Peripheral artery disease | 0.0 | 183 (15.7) | 307 (15.2) | 0.0131 | 15.7 | 15.4 | 0.0075 |
| Kidney failure | 0.0 | 68 (5.8) | 56 (2.8) | 0.1305 | 4.5 | 4.0 | 0.0248 |
| History of kidney transplant | 0.0 | 15 (1.3) | 13 (0.7) | 0.0570 | 1.2 | 0.6 | 0.0598 |
| Atrial fibrillation | 0.0 | 249 (21.4) | 436 (21.7) | 0.0063 | 21.6 | 22.0 | 0.0082 |
| Chronic obstructive pulmonary disease | 0.0 | 389 (33.4) | 610 (30.3) | 0.0664 | 32.5 | 32.3 | 0.0063 |
| Asthma | 0.0 | 147 (12.6) | 205 (10.2) | 0.0737 | 12.0 | 12.0 | 0.0001 |
| Depression | 0.0 | 655 (56.3) | 1057 (52.5) | 0.0763 | 55.4 | 55.2 | 0.0038 |
| Charlson Comorbidity Index, mean (SD) | 0.0 | 4.1 (3.3) | 3.8 (3.0) | 0.1012 | 4.0 (3.2) | 4.0 (3.1) | 0.0003 |
| ***Antihypertensive medication**** |  |  |  |  |  |  |  |
| ACEI | 0.0 | 0.0 | 2014 (100.0) | n/a | 0.0 | 100.0 | n/a |
| ARB | 0.0 | 1164 (100.0) | 0.0 | n/a | 100.0 | 0.0 | n/a |
| CCB | 0.0 | 512 (44.0) | 744 (36.9) | 0.1419 | 42.7 | 42.6 | 0.0018 |
| Thiazide diuretic | 0.0 | 293 (25.2) | 562 (27.9) | 0.0629 | 25.6 | 25.2 | 0.0081 |
| Alpha blocker | 0.0 | 124 (10.7) | 184 (9.1) | 0.0491 | 10.4 | 10.5 | 0.0033 |
| Beta blocker | 0.0 | 589 (50.6) | 877 (43.6) | 0.1411 | 49.2 | 49.4 | 0.0046 |
| Centrally acting | 0.0 | 25 (2.2) | 22 (1.1) | 0.0728 | 1.9 | 1.7 | 0.0113 |
| Direct vasodilator | 0.0 | 87 (7.5) | 76 (3.8) | 0.1407 | 5.9 | 5.8 | 0.0076 |
| Direct renin inhibitor | 0.0 | 0 (0.0) | 0 (0.0) | 0.0000 | 0.0 | 0.0 | 0.0000 |
| Aldosterone receptor antagonist | 0.0 | 81 (7.0) | 102 (5.1) | 0.0744 | 6.7 | 6.7 | 0.0007 |
| Loop diuretic | 0.0 | 254 (12.8) | 344 (17.1) | 0.1147 | 20.7 | 20.8 | 0.0028 |
| Potassium-sparing diuretic | 0.0 | 18 (1.6) | 19 (0.9) | 0.0488 | 1.3 | 1.3 | 0.0043 |
| Total number of antihypertensive medications in regimen | 0.0 | 2.7 (1.1) | 2.5 (1.1) | 0.2179 | 2.6 (1.1) | 2.6 (1.1) | 0.0027 |
| ***Other medication use*** |  |  |  |  |  |  |  |
| Current statin use | 0.0 | 802 (38.9) | 1371 (68.1) | 0.0179 | 68.7 | 68.8 | 0.0005 |
| Current aspirin use | 0.0 | 327 (28.1) | 496 (24.6) | 0.0771 | 27.5 | 27.3 | 0.0061 |
| Numbers in table are frequency (column %) or mean (SD) for before-weighting variables, and column % or mean (SD) for after-weighting variables. | | | | | | | |
| ACEI: angiotensin-converting enzyme inhibitor; ARB: angiotensin II receptor blocker; ASMD: absolute standardized mean difference; BP: blood pressure; CCB: calcium channel blocker; eGFR: estimated glomerular filtration rate; HDL-C: high-density lipoprotein cholesterol; LDL-C: low-density lipoprotein cholesterol; SD: standard deviation; USD: United States Dollars | | | | | | | |
| * Variables not included in calculation of propensity score because these variables were constructs of the exposure definitions, and therefore will not have ASMD <0.1 after weighting. | | | | | | | |

| Supplemental Table S8: Matching weight adjusted incidence rates and hazard ratios for all-cause hospitalization and all-cause mortality among outpatient Veterans with treated hypertension who are current users of an ARB/ACEI-based antihypertensive regimen vs. non-ARB/ACEI-based antihypertensive regimen in subgroups, matching weight adjusted. | | | | | |
| --- | --- | --- | --- | --- | --- |
| **Subgroup** | **ARB/ACEI-based antihypertensive regimen** | **Non-ARB/ACEI-based antihypertensive regimen** | **Hazard ratio** |  |  |
|  |  |  | **(95% CI)** | **p-value** | **P_interaction_** |
| Age, years |  |  |  |  |  |
| <65 (n=3,101) | 129  (3.1) | 190  (4.3) | 0.74  (0.59, 0.94) | 0.011 | 0.07 |
| ≥65 (n=1,868) | 185  (8.7) | 205  (9.0) | 0.99  (0.81, 1.22) | 0.94 |  |
| Sex |  |  |  |  |  |
| Male (n=4,392) | 297  (5.2) | 356  (6.3) | 0.84  (0.72, 0.98) | 0.030 | 0.76 |
| Female (n=577) | 39  (3.5) | 17  (2.7) | 1.31  (0.69, 2.52) | 0.44 |  |
| Race-ethnicity |  |  |  |  |  |
| Non-Hispanic Black (n=1,850) | 159  (5.0) | 114  (5.1) | 0.99  (0.77, 1.26) | 0.98 | 0.11 |
| All other race-ethnicities (n=3,119) | 200  (4.9) | 236  (6.7) | 0.76  (0.63, 0.90) | 0.004 |  |
| Body mass index, kg/m^2^ |  |  |  |  |  |
| Non-obese (<30) (n=1,385) | 96  (6.3) | 136  (6.7) | 0.94  (0.73, 1.24) | 0.64 | 0.71 |
| Obesity (≥30) (n=3,584) | 150  (5.2) | 157  (5.8) | 0.93  (0.73, 1.15) | 0.48 |  |
| Antihypertensive medications being taken |  |  |  |  |  |
| Less than three (n=4,194) | 203  (4.3) | 376  (6.0) | 0.74  (0.63, 0.88) | <0.001 | 0.003 |
| Three or more (n=775) | 111  (7.6) | 19  (4.8) | 1.52  (0.91, 2.71) | 0.12 |  |
| Numbers in table are expressed as frequency of event (rate per 100 person-months). | | | | | |
| ACEI: angiotensin-converting enzyme inhibitor; ARB: angiotensin II receptor blocker; CI: confidence interval | | | | | |

| Supplemental Table S9: Matching weight adjusted incidence rates and hazard ratios for all-cause hospitalization and all-cause mortality among outpatient Veterans with treated hypertension who are current users of an ARB vs. ACEI in subgroups. | | | | | |  |
| --- | --- | --- | --- | --- | --- | --- |
| **Subgroup** | **ARB user** | **ACEI user** | **Hazard ratio** |  |  |  |
|  |  |  | **(95% CI)** | **p-value** | **P_interaction_** |  |
| Age, years |  |  |  |  |  |  |
| <65 (n=5,466) | 418  (8.6) | 707  (9.2) | 0.95  (0.85, 1.07) | 0.39 | 0.21 |  |
| ≥65 (n=8,115) | 1017  (16.6) | 1760  (18.8) | 0.91  (0.85, 0.97) | 0.0068 |  |  |
| Sex |  |  |  |  |  |  |
| Male (n=12,836) | 1363  (13.7) | 2382  (15.3) | 0.91  (0.86, 0.97) | 0.0023 | 0.58 |  |
| Female (n=745) | 72  (6.7) | 85  (9.6) | 0.72  (0.52, 1.03) | 0.091 |  |  |
| Race-ethnicity |  |  |  |  |  |  |
| Non-Hispanic Black (n=4,498) | 568  (12.9) | 928  (15.7) | 0.85  (0.77, 0.93) | <0.001 | 0.036 |  |
| All other race-ethnicities (n=9,083) | 867  (13.3) | 1539  (14.3) | 0.94  (0.87, 1.02) | 0.13 |  |  |
| Body mass index, kg/m^2^ |  |  |  |  |  |  |
| Non-obese (<30) (n=3,732) | 468  (17.5) | 893  (20.3) | 0.88  (0.80, 0.97) | 0.015 | 0.39 |  |
| Obesity (≥30) (n=9,849) | 698  (13.0) | 1045  (14.2) | 0.93  (0.86, 1.02) | 0.13 |  |  |
| Antihypertensive medications being taken |  |  |  |  |  |  |
| Less than three (n=8,059) | 661  (11.0) | 1351  (12.3) | 0.91  (0.83, 0.99) | 0.030 | 0.52 |  |
| Three or more (n=5,522) | 774  (16.0) | 1116  (17.9) | 0.92  (0.84, 1.00) | 0.044 |  |  |
| Numbers in table are expressed as frequency of event (rate per 100 person-months). | | | | | |  |
| ACEI: angiotensin-converting enzyme inhibitor; ARB: angiotensin II receptor blocker; CI: confidence interval | | | | | | |

| Supplemental Table S10: Matching weight adjusted incidence rates and hazard ratios for all-cause hospitalization Veterans hospitalized for COVID-19 who are current users of an ARB vs. ACEI in subgroups. | | | | | |
| --- | --- | --- | --- | --- | --- |
| **Subgroup** | **ARB user** | **ACEI user** | **Hazard ratio** |  |  |
|  |  |  | **(95% CI)** | **p-value** | **P_interaction_** |
| Age, years |  |  |  |  |  |
| <65 (n=987) | 33  (9.4) | 32  (6.8) | 1.38  (0.81, 2.42) | 0.25 | 0.25 |
| ≥65 (n=2,191) | 135  (20.4) | 210  (18.5) | 1.10  (0.87, 1.37) | 0.39 |  |
| Sex |  |  |  |  |  |
| Male (n=3,050) | 163  (17.5) | 236  (15.2) | 1.15  (0.83, 1.40) | 0.18 | 0.76 |
| Female (n=128) | 5  (4.0) | 6  (5.0) | 1.09  (0.15, 9.13) | 0.89 |  |
| Race-ethnicity |  |  |  |  |  |
| Non-Hispanic Black (n=1,262) | 78  (19.2) | 82  (12.6) | 1.48  (1.06, 2.08) | 0.015 | 0.025 |
| All other race-ethnicities (n=1,916) | 90  (16.1) | 160  (17.4) | 0.95  (0.72, 1.24) | 0.65 |  |
| Body mass index, kg/m^2^ |  |  |  |  |  |
| Non-obese (<30) (n=1,176) | 66  (19.3) | 98  (15.3) | 1.25  (0.91, 1.73) | 0.16 | 0.58 |
| Obesity (≥30) (n=2,002) | 77  (15.9) | 102  (14.1) | 1.13  (0.81, 1.53) | 0.46 |  |
| Antihypertensive medications being taken |  |  |  |  |  |
| Less than three (n=1,669) | 72  (15.3) | 123  (15.3) | 1.01  (0.72, 1.35) | 0.99 | 0.73 |
| Three or more (1,509) | 96  (19.0) | 119  (14.8) | 1.26  (0.94, 1.70) | 0.12 |  |
| Numbers in table are expressed as frequency of event (rate per 100 person-months).  ACEI: angiotensin-converting enzyme inhibitor; ARB: angiotensin II receptor blocker; CI: confidence interval | | | | | |

| Supplemental Table S11: Hazard ratios for all-cause hospitalization and all-cause mortality among outpatient Veterans with treated hypertension, by covariate adjustment strategy. | | | | | | |
| --- | --- | --- | --- | --- | --- | --- |
| **Outcome** | **ARB/ACEI-based antihypertensive regimen vs. non-ARB/ACEI-based antihypertensive regimen comparison** | | | **ARB user vs. ACEI user comparison** | | |
|  | **Total N included** | **HR** |  | **Total N included** | **HR** |  |
|  | **in analysis** | **(95% CI)** | **p-value** | **in analysis** | **(95% CI)** | **p-value** |
| ***All-cause hospitalization or all-cause mortality*** |  |  |  |  |  |  |
| Crude | 4,969 | 0.79  (0.68, 0.91) | 0.0014 | 13,581 | 1.05  (0.98, 1.12) | 0.18 |
| Multivariable-adjusted | 4,969 | 0.87  (0.75, 1.03) | 0.12 | 13,581 | 0.91  (0.84, 0.97) | 0.0055 |
| Propensity score as covariate | 4,969 | 0.86  (0.74, 1.00) | 0.050 | 13,581 | 0.91  (0.86, 0.97) | 0.0043 |
| Propensity score stratification | 4,969 | 0.86  (0.74, 1.00) | 0.050 | 13,581 | 0.92  (0.86, 0.97) | 0.0055 |
| Propensity score matching | 4,092 | 0.84  (0.71, 0.99) | 0.038 | 9,584 | 0.92  (0.86, 0.98) | 0.011 |
| IPTW | 4,969 | 0.87  (0.75, 1.00) | 0.07 | 13,581 | 0.92  (0.87, 1.98) | 0.0083 |
| Matching weight adjusted (primary analysis) | 4,969 | 0.85  (0.73, 0.99) | 0.035 | 13,581 | 0.91  (0.86, 0.97) | 0.0020 |
| ***Secondary Outcomes*** |  |  |  |  |  |  |
| All-cause hospitalization |  |  |  |  |  |  |
| Crude | 4,969 | 0.79  (0.67, 0.92) | 0.0030 | 13,581 | 1.05  (0.98, 1.13) | 0.17 |
| Multivariable-adjusted | 4,969 | 0.89  (0.75, 1.06) | 0.24 | 13,581 | 0.91  (0.85, 0.98) | 0.018 |
| Propensity score as covariate | 4,969 | 0.88  (0.75, 1.02) | 0.10 | 13,581 | 0.92  (0.86, 0.98) | 0.011 |
| Propensity score stratification | 4,969 | 0.87  (0.75, 1.02) | 0.10 | 13,581 | 0.92  (0.86, 0.98) | 0.014 |
| Propensity score matching | 4,092 | 0.85  (0.71, 1.01) | 0.13 | 9,584 | 0.92  (0.85, 0.99) | 0.023 |
| IPTW | 4,969 | 0.89  (0.76, 1.03) | 0.16 | 13,581 | 0.93  (0.87, 0.99) | 0.021 |
| Matching weight adjusted (primary analysis) | 4,969 | 0.86  (0.73, 1.01) | 0.07 | 13,581 | 0.91  (0.85, 0.97) | 0.0052 |
| All-cause mortality |  |  |  |  |  |  |
| Crude | 4,969 | 0.96  (0.66, 1.39) | 0.76 | 13,581 | 1.11  (0.97, 1.26) | 0.12 |
| Multivariable-adjusted | 4,969 | 0.92  (0.58, 1.43) | 0.75 | 13,581 | 0.94  (0.82, 1.08) | 0.38 |
| Propensity score as covariate | 4,969 | 0.94  (0.65, 1.37) | 0.78 | 13,581 | 0.95  (0.84, 1.08) | 0.46 |
| Propensity score stratification | 4,969 | 0.94  (0.65, 1.39) | 0.76 | 13,581 | 0.96  (0.84, 1.08) | 0.48 |
| Propensity score matching | 4,092 | 0.95  (0.63, 1.42) | 0.99 | 9,584 | 0.96  (0.84, 1.11) | 0.58 |
| IPTW | 4,969 | 0.94  (0.65, 1.38) | 0.76 | 13,581 | 0.95  (0.84, 1.07) | 0.39 |
| Matching weight adjusted (primary analysis) | 4,969 | 0.95  (0.66, 1.36) | 0.77 | 13,581 | 0.95  (0.84, 1.08) | 0.44 |
| ICU admission |  |  |  |  |  |  |
| Crude | 4,969 | 1.26  (0.87, 1.84) | 0.23 | 13,581 | 0.93  (0.80, 1.09) | 0.37 |
| Multivariable-adjusted | 4,969 | 1.43  (0.94, 2.20) | 0.11 | 13,581 | 0.87  (0.74, 1.03) | 0.10 |
| Propensity score as covariate | 4,969 | 1.37  (0.92, 2.05) | 0.12 | 13,581 | 0.84  (0.72, 0.98) | 0.027 |
| Propensity score stratification | 4,969 | 1.37  (0.92, 2.05) | 0.13 | 13,581 | 0.84  (0.72, 0.98) | 0.029 |
| Propensity score matching | 4,092 | 1.29  (0.86, 1.96) | 0.36 | 9,584 | 0.84  (0.71, 1.00) | 0.051 |
| IPTW | 4,969 | 1.39  (0.95, 2.02) | 0.09 | 13,581 | 0.85  (0.72, 0.99) | 0.033 |
| Matching weight adjusted (primary analysis) | 4,969 | 1.29  (0.87, 1.92) | 0.21 | 13,581 | 0.84  (0.71, 0.98) | 0.024 |
| ACEI: angiotensin-converting enzyme inhibitor; ARB: angiotensin II receptor blocker; CI: confidence interval; ICU: intensive care unit; HR: hazard ratio; IPTW: inverse propensity treatment weighting | | | | | | |

| Supplemental Table S12: Hazard ratios for the primary and secondary outcomes among inpatient Veterans with treated hypertension, by covariate adjustment strategy. | | | | | | |
| --- | --- | --- | --- | --- | --- | --- |
| **Outcome** | **ARB/ACEI- vs. non-ARB/ACEI-based antihypertensive regimen comparison** | | | **ARB user vs. ACEI user comparison** | | |
|  | **Total N included** | **HR** |  | **Total N included** | **HR** |  |
|  | **in analysis** | **(95% CI)** | **p-value** | **in analysis** | **(95% CI)** | **p-value** |
| ***All-cause mortality*** |  |  |  |  |  |  |
| Crude | 485 | 1.21  (0.37, 3.96) | 0.75 | 3178 | 1.23  (1.01, 1.50) | 0.039 |
| Multivariable-adjusted | n/a | n/a | n/a | 3178 | 1.15  (0.92, 1.44) | 0.23 |
| Propensity score as covariate | 485 | 1.18  (0.35, 3.97) | 0.78 | 2090 | 1.14  (0.94, 1.38) | 0.20 |
| Propensity score stratification | 485 | 1.19  (0.33, 4.31) | 0.79 | 3178 | 1.14  (0.94, 1.39) | 0.19 |
| Propensity score matching | 336 | 1.44  (0.03, 71.89) | 0.85 | 3178 | 1.14  (0.91, 1.42) | 0.26 |
| IPTW | 485 | 1.09  (0.31, 3.82) | 0.89 | 3178 | 1.15  (0.94, 1.39) | 0.18 |
| Matching weight adjusted (primary analysis) | 485 | 1.25  (0.30, 5.13) | 0.76 | 3178 | 1.13  (0.93, 1.38) | 0.23 |
| ***Secondary Outcomes*** |  |  |  |  |  |  |
| ICU admission |  |  |  |  |  |  |
| Crude | 485 | 1.34  (0.77, 2.32) | 0.31 | 3178 | 0.91  (0.78, 1.05) | 0.20 |
| Multivariable-adjusted | 485 | 0.82  (0.01, 47.9) | 0.92 | 3178 | 0.92  (0.78, 1.08) | 0.31 |
| Propensity score as covariate | 485 | 1.01  (0.43, 1.89) | 0.97 | 2090 | 0.92  (0.79, 1.07) | 0.30 |
| Propensity score stratification | 485 | 1.02  (0.53, 1.97) | 0.95 | 3178 | 0.92  (0.79, 1.08) | 0.31 |
| Propensity score matching | 336 | 1.11  (0.55, 2.24) | 0.78 | 3178 | 0.92  (0.77, 1.10) | 0.36 |
| IPTW | 485 | 1.05  (0.54, 2.04) | 0.88 | 3178 | 0.93  (0.79, 1.08) | 0.32 |
| Matching weight adjusted (primary analysis) | 485 | 1.05  (0.54, 1.94) | 0.94 | 3178 | 0.92  (0.79, 1.08) | 0.29 |
| Dialysis |  |  |  |  |  |  |
| Crude | n/a | n/a | n/a | 3054 | 1.77  (1.34, 2.35) | <0.001 |
| Multivariable-adjusted | n/a | n/a | n/a | 3054 | 1.00  (0.71, 1.41) | 0.99 |
| Propensity score as covariate | n/a | n/a | n/a | 3054 | 1.06  (0.82, 1.38) | 0.64 |
| Propensity score stratification | n/a | n/a | n/a | 3054 | 1.14  (0.87, 1.48) | 0.34 |
| Propensity score matching | n/a | n/a | n/a | 2090 | 1.15  (0.86, 1.54) | 0.34 |
| IPTW | n/a | n/a | n/a | 3054 | 1.04  (0.81, 1.34) | 0.74 |
| Matching weight adjusted (primary analysis) | n/a | n/a | n/a | 3054 | 1.08  (0.84, 1.40) | 0.54 |
| Mechanical ventilation |  |  |  |  |  |  |
| Crude | 485 | 0.73  (0.31, 1.76) | 0.49 | 3151 | 1.03  (0.86, 1.23) | 0.77 |
| Multivariable-adjusted | n/a | n/a | n/a | 3151 | 0.97  (0.79, 1.18) | 0.76 |
| Propensity score as covariate | 485 | 0.70  (0.27, 1.79) | 0.45 | 3151 | 0.97  (0.81, 1.16) | 0.73 |
| Propensity score stratification | 485 | 0.70  (0.26, 1.92) | 0.49 | 3151 | 0.97  (0.81, 1.16) | 0.73 |
| Propensity score matching | 336 | 0.67  (0.22, 2.03) | 0.47 | 2090 | 0.96  (0.79, 1.18) | 0.71 |
| IPTW | 485 | 0.68  (0.25, 1.84) | 0.45 | 3151 | 0.98  (0.82, 1.17) | 0.83 |
| Matching weight adjusted (primary analysis) | 485 | 0.67  (0.22, 2.03) | 0.47 | 3151 | 0.96  (0.80, 1.15) | 0.65 |
| *Cannot be reported due to small sample size and errors with bootstrapping.  ACEI: angiotensin-converting enzyme inhibitor; ARB: angiotensin II receptor blocker; CI: confidence interval; ICU: intensive care unit; HR: hazard ratio; IPTW: inverse propensity treatment weighting | | | | | | |

| Supplemental Table S13: Matching weight adjusted incidence rates and hazard ratios for the primary and secondary outcomes among outpatient Veterans with treated hypertension who are current users of an ARB/ACEI-based antihypertensive regimen vs. non-ARB/ACEI-based antihypertensive regimen, by varying the definition of the primary medication exposure. | | | | |
| --- | --- | --- | --- | --- |
| **Outcome** | **ARB/ACEI-based antihypertensive regimen** | **Non-ARB/ACEI-based antihypertensive regimen** | **Hazard ratio** |  |
|  | **N event/N exposed (Rate per 100 person-years)** | **N event/N exposed**  **(Rate per 100 person-years)** | **(95% CI)** | **p-value** |
| ***All-cause hospitalization or all-cause mortality*** |  |  |  |  |
| Primary analysis | 314 / 2482  (5.0) | 395 / 2487  (6.0) | 0.85  (0.73, 0.99) | 0.035 |
| Alternative definition 1 | 261 / 2090  (4.8) | 326 / 2068  (6.1) | 0.82  (0.69, 0.96) | 0.017 |
| Alternative definition 2 | 231 / 1766  (5.2) | 289 / 1808  (6.2) | 0.85  (0.72, 1.02) | 0.08 |
| Alternative definition 3 | 276 / 2164  (5.1) | 349 / 2175  (6.1) | 0.85  (0.73, 1.00) | 0.05 |
| ***Secondary Outcomes*** |  |  |  |  |
| All-cause hospitalization |  |  |  |  |
| Primary analysis | 273 / 2482  (4.3) | 344 / 2487  (5.1) | 0.86  (0.73, 1.01) | 0.07 |
| Alternative definition 1 | 226 / 2090  (4.1) | 282 / 2068  (5.1) | 0.84  (0.70, 1.00) | 0.05 |
| Alternative definition 2 | 199 / 1766  (4.4) | 250 / 1808  (5.2) | 0.86  (0.71, 1.05) | 0.13 |
| Alternative definition 3 | 242 / 2164  (4.4) | 306 / 2175  (5.2) | 0.87  (0.73, 1.03) | 0.10 |
| All-cause mortality |  |  |  |  |
| Primary analysis | 61 / 2482  (0.8) | 65 / 2487  (0.9) | 0.95  (0.66, 1.36) | 0.78 |
| Alternative definition 1 | 52 / 2090  (0.8) | 57 / 2068  (0.9) | 0.87  (0.59, 1.30) | 0.50 |
| Alternative definition 2 | 46 / 1766  (0.9) | 49 / 1808  (1.0) | 0.96  (0.63, 1.46) | 0.85 |
| Alternative definition 3 | 49 / 2164  (0.8) | 56 / 2175  (0.9) | 0.87  (0.58, 1.31) | 0.52 |
| ICU admission |  |  |  |  |
| Primary analysis | 61 / 2482  (0.9) | 49 / 2487  (0.7) | 1.29  (0.87, 1.92) | 0.21 |
| Alternative definition 1 | 48 / 2090  (0.8) | 49 / 2068  (0.7) | 1.13  (0.73, 1.74) | 0.59 |
| Alternative definition 2 | 46 / 1766  (1.0) | 40 / 1808  (0.8) | 1.21  (0.77, 1.90) | 0.40 |
| Alternative definition 3 | 55 / 2164  (0.9) | 47 / 2175  (0.8) | 1.22  (0.81, 1.84) | 0.34 |
| Numbers in table are expressed as unweighted frequency of event (weighted rate per 100 person-months).  Primary analysis: Veteran was considered exposed if they had a pharmacy fill for the medication class within 90 days (+14 days) prior to the index date (including the index date).  Alternative definition 1: Veteran was considered exposed if they met primary analysis definition AND had ≥1 pharmacy fills for the medication class in each of the two, six-month periods during the 365 days (+14 days) prior to the index date (including the index date). The pharmacy fills do not have to be consecutive.  Alternative definition 2: Veteran was considered exposed if they met primary analysis definition AND had ≥1 pharmacy fill in the 90 days (+14 days) prior to the index date (including the index date) for the medication class and 3 consecutive pharmacy fills for the same medication class in the 1 year (+ 14 days) prior to the index date (including the index date). A “consecutive” fill is defined as pharmacy fills with less than 30 days of gap in days’ supply between the fills.  Alternative definition 3: Veteran was considered exposed if they met primary analysis definition AND had a pharmacy fill dispensed before the index date with a day supply that met or exceeded the index date. | | | | |
| ACEI: angiotensin-converting enzyme inhibitor; ARB: angiotensin II receptor blocker; CI: confidence interval; EHR: electronic health record; ICU: intensive care unit; IPTW: inverse propensity treatment weighting | | | | |

| Supplemental Table S14: Matching weight adjusted incidence rates and hazard ratios for the primary and secondary outcomes among outpatient Veterans with treated hypertension who are current users of an ARB vs. ACEI, by varying the definition of the primary medication exposure. | | | | |
| --- | --- | --- | --- | --- |
| **Outcome** | **ARB user** | **ACEI user** | **Hazard ratio** |  |
|  | **N event/N exposed (Rate per 100 person-years)** | **N event/N exposed**  **(Rate per 100 person-years)** | **(95% CI)** | **p-value** |
| ***All-cause hospitalization or all-cause mortality*** |  |  |  |  |
| Primary analysis | 1435 / 4877  (13.2) | 2467 / 8704  (14.8) | 0.91  (0.86, 0.97) | 0.002 |
| Alternative definition 1 | 1216 / 4208  (13.0) | 2067 / 7434  (14.6) | 0.91  (0.85, 0.97) | 0.004 |
| Alternative definition 2 | 1126 / 3858  (13.5) | 1830 / 6406  (15.3) | 0.90  (0.85, 0.96) | 0.002 |
| Alternative definition 3 | 1219 / 4097  (13.0) | 2167 / 7769  (14.6) | 0.90  (0.84, 0.97) | 0.003 |
| ***Secondary Outcomes*** |  |  |  |  |
| All-cause hospitalization |  |  |  |  |
| Primary analysis | 1254 / 4877  (10.9) | 2146 / 8704  (12.3) | 0.91  (0.85, 0.97) | 0.005 |
| Alternative definition 1 | 1060 / 4208  (10.8) | 1802 / 7434  (12.1) | 0.90  (0.84, 0.97) | 0.004 |
| Alternative definition 2 | 983 / 3858  (11.2) | 1601 / 6406  (12.7) | 0.89  (0.83, 0.96) | 0.002 |
| Alternative definition 3 | 1067 / 4097  (10.8) | 1905 / 7769  (12.2) | 0.90  (0.84, 0.96) | 0.003 |
| All-cause mortality |  |  |  |  |
| Primary analysis | 386 / 4877  (2.7) | 628 / 8704  (2.8) | 0.95  (0.84, 1.08) | 0.44 |
| Alternative definition 1 | 324 / 4208  (2.7) | 515 / 7434  (2.7) | 0.97  (0.84, 1.12) | 0.70 |
| Alternative definition 2 | 298 / 3858  (2.7) | 460 / 6406  (2.8) | 0.98  (0.84, 1.13) | 0.74 |
| Alternative definition 3 | 329 / 4097  (2.7) | 524 / 7769  (2.7) | 0.98  (0.86, 1.13) | 0.82 |
| ICU admission |  |  |  |  |
| Primary analysis | 257 / 4877  (1.8) | 491 / 8704  (2.1) | 0.84  (0.71, 0.98) | 0.024 |
| Alternative definition 1 | 216 / 4208  (1.7) | 402 / 7434  (2.0) | 0.85  (0.72, 1.00) | 0.05 |
| Alternative definition 2 | 207 / 3858  (1.9) | 372 / 6406  (2.2) | 0.85  (0.72, 1.01) | 0.07 |
| Alternative definition 3 | 226 / 4097  (1.8) | 435 / 7769  (2.1) | 0.86  (0.74, 1.01) | 0.07 |
| Numbers in table are expressed as unweighted frequency of event (weighted rate per 100 person-months).  Primary analysis: Veteran was considered exposed if they had a pharmacy fill for the medication class within 90 days (+14 days) prior to the index date (including the index date).  Alternative definition 1: Veteran was considered exposed if they met primary analysis definition AND had ≥1 pharmacy fills for the medication class in each of the two, six-month periods during the 365 days (+14 days) prior to the index date (including the index date). The pharmacy fills do not have to be consecutive.  Alternative definition 2: Veteran was considered exposed if they met primary analysis definition AND had ≥1 pharmacy fill in the 90 days (+14 days) prior to the index date (including the index date) for the medication class and 3 consecutive pharmacy fills for the same medication class in the 1 year (+ 14 days) prior to the index date (including the index date). A “consecutive” fill is defined as pharmacy fills with less than 30 days of gap in days’ supply between the fills.  Alternative definition 3: Veteran was considered exposed if they met primary analysis definition AND had a pharmacy fill dispensed before the index date with a day supply that met or exceeded the index date. | | | | |
| ACEI: angiotensin-converting enzyme inhibitor; ARB: angiotensin II receptor blocker; CI: confidence interval; EHR: electronic health record; ICU: intensive care unit; IPTW: inverse propensity treatment weighting | | | | |

| Supplemental Table S15: Matching weight adjusted incidence rates and hazard ratios for the primary and secondary outcomes among inpatient Veterans with treated hypertension who are current users of an ARB vs. ACEI, by varying the definition of the primary medication exposure. | | | | |
| --- | --- | --- | --- | --- |
| **Outcome** | **ARB user** | **ACEI user** | **Hazard ratio** |  |
|  | **N event/N exposed (Rate per 100 person-years)** | **N event/N exposed**  **(Rate per 100 person-years)** | **(95% CI)** | **p-value** |
| ***All-cause mortality*** |  |  |  |  |
| Primary analysis | 168 / 1164  (21.0) | 242 / 2014  (17.7) | 1.13  (0.93, 1.38) | 0.23 |
| Alternative definition 1 | 136 / 973  (16.6) | 193 / 1692  (13.7) | 1.19  (0.96, 1.49) | 0.12 |
| Alternative definition 2 | 129 / 907  (17.1) | 184 / 1496  (14.6) | 1.14  (0.90, 1.44) | 0.27 |
| Alternative definition 3 | 144 / 982  (17.0) | 211 / 1785  (15.3) | 1.11  (0.89, 1.37) | 0.36 |
| Alternative definition 4 | 71 / 930  (8.4) | 119 / 1618  (8.5) | 0.96  (0.70, 1.32) | 0.79 |
| ***Secondary Outcomes*** |  |  |  |  |
| ICU admission |  |  |  |  |
| Primary analysis | 264 / 1164  (26.4) | 497 / 2014  (24.5) | 0.92  (0.79, 1.08) | 0.29 |
| Alternative definition 1 | 226 / 973  (32.8) | 407 / 1692  (33.8) | 0.96  (0.81, 1.14) | 0.63 |
| Alternative definition 2 | 212 / 907  (33.5) | 372 / 1496  (34.3) | 0.95  (0.80, 1.14) | 0.60 |
| Alternative definition 3 | 231 / 982  (32.8) | 444 / 1785  (34.8) | 0.94  (0.80, 1.11) | 0.47 |
| Alternative definition 4 | 181 / 930  (26.2) | 357 / 1618  (30.1) | 0.86  (0.71, 1.04) | 0.12 |
| Dialysis |  |  |  |  |
| Primary analysis | 33 / 1164  (4.6) | 448 / 2014  (4.5) | 1.08  (0.84, 1.40) | 0.54 |
| Alternative definition 1 | 27 / 973  (3.3) | 40 / 1692  (3.6) | 1.14  (0.86, 1.52) | 0.36 |
| Alternative definition 2 | 24 / 907  (3.2) | 32 / 1496  (3.3) | 1.09  (0.80, 1.49) | 0.57 |
| Alternative definition 3 | 31 / 982  (3.7) | 43 / 1785  (4.0) | 1.03  (0.77, 1.37) | 0.86 |
| Alternative definition 4 | 13 / 930  (1.8) | 24 / 1618  (2.3) | 1.15  (0.81, 1.62) | 0.43 |
| Mechanical ventilation |  |  |  |  |
| Primary analysis | 185 / 1164  (24.5) | 313 / 2014  (23.9) | 0.96  (0.80, 1.15) | 0.65 |
| Alternative definition 1 | 151 / 973  (20.4) | 258 / 1692  (20.9) | 0.95  (0.78, 1.16) | 0.62 |
| Alternative definition 2 | 136 / 907  (19.9) | 230 / 1496  (21.1) | 0.94  (0.76, 1.16) | 0.55 |
| Alternative definition 3 | 162 / 982  (21.2) | 276 / 1785  (22.3) | 0.95  (0.78, 1.15) | 0.60 |
| Alternative definition 4 | 97 / 930  (12.8) | 182 / 1618  (14.7) | 0.88  (0.68, 1.12) | 0.30 |
| Numbers in table are expressed as unweighted frequency of event (weighted rate per 100 person-months).  Primary analysis: Veteran was considered exposed if they had a pharmacy fill for the medication class within 90 days (+14 days) prior to the index date (including the index date).  Alternative definition 1: Veteran was considered exposed if they met primary analysis definition AND had ≥1 pharmacy fills for the medication class in each of the two, six-month periods during the 365 days (+14 days) prior to the index date (including the index date). The pharmacy fills do not have to be consecutive.  Alternative definition 2: Veteran was considered exposed if they met primary analysis definition AND had ≥1 pharmacy fill in the 90 days (+14 days) prior to the index date (including the index date) for the medication class and 3 consecutive pharmacy fills for the same medication class in the 1 year (+ 14 days) prior to the index date (including the index date). A “consecutive” fill is defined as pharmacy fills with less than 30 days of gap in days’ supply between the fills.  Alternative definition 3: Veteran was considered exposed if they met primary analysis definition AND had a pharmacy fill dispensed before the index date with a day supply that met or exceeded the index date.  Alternative exposure definition 4: Veteran was considered exposed if they met primary analysis definition AND had ≥1 medication administration for the same antihypertensive medication after the index date (including the index date). | | | | |
| ACEI: angiotensin-converting enzyme inhibitor; ARB: angiotensin II receptor blocker; CI: confidence interval; EHR: electronic health record; ICU: intensive care unit; IPTW: inverse propensity treatment weighting | | | | |

| Supplemental Table S16: One or more inpatient encounter for negative control outcomes among outpatient Veterans with treated hypertension, matching weight adjusted. | | | | |
| --- | --- | --- | --- | --- |
| **Negative control outcome** | **ARB/ACEI-based antihypertensive regimen** | **Non-ARB/ACEI-based antihypertensive regimen** | **Matching weight-adjusted HR** |  |
|  | **(n = 2,482)** | **(n = 2,487)** | **(95% CI)** | **p-value** |
| Severe gastrointestinal bleeding or urinary tract infection | 31  (0.5) | 45  (0.5) | 0.89  (0.55, 1.43) | 0.63 |
|  | **ARB user** | **ACEI user** | **Matching weight-adjusted HR** |  |
|  | **(n = 4,877)** | **(n = 8,704)** | **(95% CI)** | **p-value** |
| Severe gastrointestinal bleeding or urinary tract infection | 214  (1.4) | 351  (1.5) | 0.97  (0.81, 1.14) | 0.74 |
| Numbers in table are expressed as frequency of event (rate per 100 person-months).  ACEI: angiotensin-converting enzyme inhibitor; ARB: angiotensin II receptor blocker; CI: confidence interval; HR: hazard ratio | | | | |

| Supplemental Table S17: One or more inpatient encounter for negative control outcomes among inpatient Veterans with treated hypertension, matching weight adjusted. | | | | |
| --- | --- | --- | --- | --- |
|  | **ARB/ACEI-based antihypertensive regimen** | **Non-ARB/ACEI-based antihypertensive regimen** | **Matching weight-adjusted HR** |  |
|  | **(n = 210)** | **(n = 275)** | **(95% CI)** | **p-value** |
| Severe gastrointestinal bleeding or urinary tract infection | 18  (3.4) | 20  (1.6) | 2.00  (0.94, 4.51) | 0.07 |
|  | **ARB user** | **ACEI user** | **Matching weight-adjusted HR** |  |
|  | **(n = 1,164)** | **(n = 2,014)** | **(95% CI)** | **p-value** |
| Severe gastrointestinal bleeding or urinary tract infection | 183  (5.6) | 298  (5.3) | 1.04  (0.85, 1.26) | 0.68 |
| Numbers in table are expressed as frequency of event (rate per 100 person-months).  ACEI: angiotensin-converting enzyme inhibitor; ARB: angiotensin II receptor blocker; CI: confidence interval; HR: hazard ratio | | | | |

Supplemental Figures

Supplemental Figure S1: Index date identification period and collection of variables in outpatients (Panel A) and inpatients (Panel B).

Panel A:

14 day grace period allowed for all medication exposures to account for potential non-adherence.

Panel B:

14 day grace period allowed for all medication exposures to account for potential non-adherence.

Supplemental Figure S2: Distribution of propensity scores among outpatient Veterans who are SARS-CoV-2 positive between current users of an ARB/ACEI-based antihypertensive regimen vs. non-ARB/ACEI-based antihypertensive regimen (Panel A), and current users of an ARB vs. ACEI (Panel B).

Panel A:

Panel B:

Supplemental Figure S3: Distribution of propensity scores among inpatient Veterans between current users of an ARB/ACEI-based antihypertensive regimen vs. non-ARB/ACEI-based antihypertensive regimen (Panel A) and current users of an ARB vs. ACEI (Panel B).

Panel A:

Panel B:

Supplemental Figure S4: Flow diagrams for inclusion of outpatient (Panel A) and inpatient (Panel B) Veterans.

Panel A:

Panel B:

**Supplemental Figure S5: Balance of patient characteristics before and after propensity score weighting.**


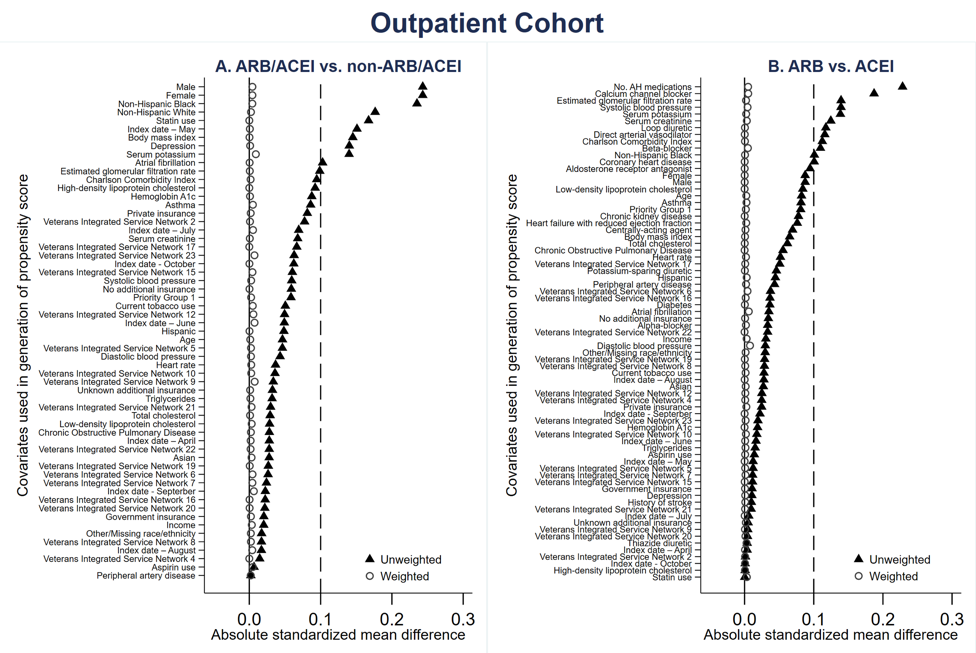


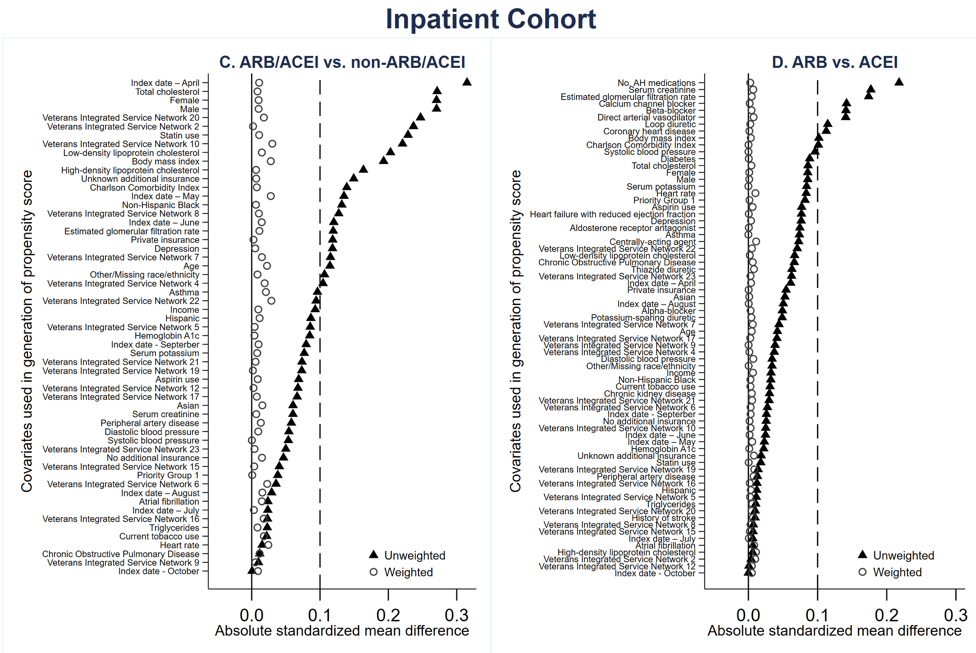


Propensity score weighting reduced ASMDs in baseline characteristics between exposure groups to <0.1 in outpatients (Panels A and B) and inpatients (Panels C and D). Weighted ASMDs <0.1 indicate that the weighted exposure groups have adequately small differences between measured baseline characteristics and therefore may be appropriate for inferential analysis. Shaded triangles represent unweighted ASMD values and circles represent weighted ASMD values. *Abbreviations:* ACEI: angiotensin-converting enzyme inhibitor; ARB: angiotensin II receptor blocker; ASMD: absolute standardized mean difference.

Supplemental Figure S6: Matching weight-adjusted cumulative hazard curves for all-cause hospitalization, all-cause mortality, and ICU admission among outpatient Veterans who are current users of an ARB/ACEI-based antihypertensive regimen vs. non-ARB/ACEI-based antihypertensive regimen (Panels A through C) and current users of an ARB vs. ACEI (Panels D through F) who are SARS-CoV-2 positive.

Supplemental Figure S7: Matching weight-adjusted cumulative hazard curves for ICU admission, mechanical ventilation, and dialysis among inpatient Veterans who are current users of an ARB/ACEI-based antihypertensive regimen vs. non-ARB/ACEI-based antihypertensive regimen (Panels A and B) and current users of an ARB vs. ACEI (Panels C through E) who are SARS-CoV-2 positive.

Patients were excluded from the analysis of dialysis if they had a history of ESRD (see Supplemental Table S1 for definition). Patients were excluded from the analysis of mechanical ventilation if they had a history of ventilation more than 7 days prior to their index date.

References

1. Imbens GW. Nonparametric estimation of average treatment effects under exogeneity: A review. Rev. Econ. Stat. 2004;86:4–29.

2. Efron B, Tibshirani RJ. An Introduction to the Bootstrap. CRC Press; 1994.

3. White IR, Royston P, Wood AM. Multiple imputation using chained equations: Issues and guidance for practice. Stat. Med. 2011;30:377–399.

4. Rubin DB, Schenker N. Multiple imputation in health-care databases: an overview and some applications. Stat. Med. 1991;10:585–598.

5. Quan H, Khan N, Hemmelgarn BR, et al. Validation of a case definition to define hypertension using administrative data. Hypertension 2009;54:1423–1428.

6. US Department of Veterans Affairs. Veterans Benefits Administration - Regional Offices. 2020. Available at: https://www.benefits.va.gov/benefits/offices.asp. Accessed March 13, 2020.

7. Hall RK, Wang V, Jackson GL, et al. Implementation of automated reporting of estimated glomerular filtration rate among Veterans Affairs laboratories: A retrospective study. BMC Med. Inform. Decis. Mak. 2012;12:69.

8. Vlasschaert MEO, Bejaimal SAD, Hackam DG, et al. Validity of administrative database coding for kidney disease: A systematic review. Am. J. Kidney Dis. 2011;57:29–43. Available at: http://dx.doi.org/10.1053/j.ajkd.2010.08.031.

9. Muntner P, Gutiérrez OM, Zhao H, et al. Validation study of medicare claims to identify older US adults with CKD using the reasons for geographic and racial differences in stroke (REGARDS) study. Am. J. Kidney Dis. 2015;65:249–258.

10. Kent ST, Safford MM, Zhao H, et al. Optimal Use of Available Claims to Identify a Medicare Population Free of Coronary Heart Disease. Am. J. Epidemiol. 2015;182:808–819.

11. Kumamaru H, Judd SE, Curtis JR, et al. Validity of Claims-Based Stroke Algorithms in Contemporary Medicare Data: REGARDS Study Linked with Medicare Claims. Circ. Cardiovasc. Qual. Outcomes 2014;7:611–619. Available at: http://circoutcomes.ahajournals.org/lookup/suppl/doi:10.1161/CIRCOUTCOMES.113.000743/-/DC1. Accessed November 13, 2019.

12. Jensen PN, Johnson K, Floyd J, Heckbert SR, Carnahan R, Dublin S. A systematic review of validated methods for identifying atrial fibrillation using administrative data. Pharmacoepidemiol. Drug Saf. 2012;21 Suppl 1:141–147.

13. Gothe H, Rajsic S, Vukicevic D, et al. Algorithms to identify COPD in health systems with and without access to ICD coding: A systematic review. BMC Health Serv. Res. 2019;19:737.

14. Gershon AS, Wang C, Guan J, Vasilevska-Ristovska J, Cicutto L, To T. Identifying Patients with Physician-Diagnosed Asthma in Health Administrative Databases. Can. Respir. J. 2009;16:183–188.

15. Quan H, Sundararajan V, Halfon P, et al. Coding algorithms for defining comorbidities in ICD-9-CM and ICD-10 administrative data. Med. Care 2005;43:1130–1139.

16. Quan H, Sundarajan V, Halfon P, et al. Coding Algorithms for Defining Comorbidities in ICD-9-CM and ICD-10 Administrative Data. Med. Care 2005;43:1130–1139.

17. Abraham NS, Cohen DC, Rivers B, Richardson P. Validation of administrative data used for the diagnosis of upper gastrointestinal events following nonsteroidal anti-inflammatory drug prescription. Aliment. Pharmacol. Ther. 2006;24:299–306.
